# Biosynthetic Microcin J25 Exerts Strong Antibacterial, Anti-Inflammatory Activities, Low Cytotoxicity Without Increasing Drug-Resistance to Bacteria Target

**DOI:** 10.3389/fimmu.2022.811378

**Published:** 2022-02-18

**Authors:** Haitao Yu, Lijun Shang, Guangxin Yang, Ziqi Dai, Xiangfang Zeng, Shiyan Qiao

**Affiliations:** ^1^ State Key Laboratory of Animal Nutrition, Ministry of Agriculture and Rural Affairs Feed Industry Center, China Agricultural University, Beijing, China; ^2^ Department of Immunology, Beijing Key Laboratory of Tumor Systems Biology, Institute of Systems Biomedicine, School of Basic Medical Sciences, Peking University Health Science Center, Beijing, China; ^3^ Beijing Key Laboratory of Biofeed Additives, China Agricultural University, Beijing, China

**Keywords:** antimicrobial peptide microcin J25, multidrug resistance, antimicrobial activity, mode of action, cytotoxicity, bacterial infection, inflammation

## Abstract

Multidrug resistant (MDR) bacterial infection has emerged, raising concerns about untreatable infections, and posing the highest health risks. Antimicrobial peptides (AMPs) are thought to be the best remedy for this problem. Here, we showed biosynthetic microcin J25 (MccJ25) exhibited excellent bactericidal activity against standard and clinically relevant veterinary MDR strains with high stability, no cytotoxicity, and no increase in drug resistance. Analysis of antimicrobial mechanism possessed by sensitive enterotoxigenic *Escherichia coli* (ETEC) based on electron microscopy and Sytox Green methods was carried out. Results showed excellent activity against ETEC was due to permeabilizing bacterial membranes and strong affinity. MccJ25 exhibited high endotoxin-neutralizing activity in both *in vivo* and *in vitro* environments, and mice exposed to lipopolysaccharide (LPS) showed decreased plasma LPS levels and improved survival after administration of MccJ25. In an LPS-treated mouse septicemia model, MccJ25 treatment significantly alleviated inflammatory responses by inhibiting proinflammatory factor secretion and expression. In a mouse *E. coli* infection model, administration of MccJ25 effectively improved host defense against clinically source cocktail of multidrug-resistant *E. coli* strains induced intestinal inflammation and bacteria dissemination. Results of studies on anti-inflammatory mechanisms showed that MccJ25 downregulated nuclear factor kappa B kinase and mitogen-activated protein kinase, thereby reducing the production of toll-like receptor 4, myeloid differentiation factor 88 and decreasing the key proinflammatory cytokines. These findings clarify MccJ25 may be an ideal antibacterial/antiendotoxic drug candidate that has the potential to further guide the development of anti-inflammatory and/or antimicrobial agents in the war against MDR bacterial infection.

## Introduction

It is no exaggeration to say that antibiotics have rewriting the history of public health in the past few decades (1). To date, in the postantibiotic era, the increase in antibiotic resistance has become one of the most serious health problems worldwide, so the misuse of antibiotics has led to an increasing rate of failure in the treatment of various infectious diseases (2–5). Bacterial infections in the postantibiotic era, especially multidrug-resistant (MDR) strains, are becoming the biggest killers. For instance, food, water, feed, the environment, animals, and even humans are easily infected and circulated by pathogenic bacteria (6, 7). The problem of bacterial resistance, if not addressed in time, will lead to a return to the preantibiotic era, where life is fragile, a common disease could be fatal, wound, infection and deaths can be induced easily. To catch more attention to this issue, in the quest for novel alternatives, new strategies are urgently needed to tackle the problem of antibiotic resistance. New types of antibacterial alternatives to antibiotics and strategies urgently need to be identified.

Microcins are promising antimicrobials with the potential to be applied in the food, health and veterinary (8). Microcins, a class II bacteriocin, have received attractive attention as antibacterial and anti-inflammatory agents in clinical, food and veterinary medicine use. Antimicrobial peptides (AMPs), the production of microcins, originate from gram-negative microorganisms and belong to a defense mechanism against invasion by microbial pathogens, and they are a key basis for the future development of antibiotic drugs designed to treat drug-resistant infections (9–13). Because microcins are a class of ribosomally synthesized AMPs, tissues encoded by genetic systems have conserved characteristics. Selected microcins have been widely used and studied because of their unique physiological and biochemical structures, mechanisms of action, high levels of antimicrobial activity, and immune capabilities (14–17). Most of the previous studies are still focused on microcins, elucidating how enteric bacteria exploit the efficient and subtle properties of microcins to dominate the growth in complex bacterial communities (18–20). Therefore, microcins, as natural peptides, may be good antibacterial drugs. Nature microcin J25 is composed of 21 amino acids, the sequence of MccJ25 is GGAGHVPEYFVGIGTPISFYG. Additionally, MccJ25 is a low molecular weight (2107 Da), plasmid-encoded, ribosomally synthesized AMP originally isolated from a fecal strain of *Escherichia coli* (*E. coli*). It has attracted considerable research interest because of its stable lasso structure and ribosomal encoding system, which can be combined with genetic engineering to exert powerful biological activity (21–23).

To a great extent, the comprehensive antimicrobial activity of biosynthetic AMP microcin J25 (MccJ25) against clinically source MDR *E. coli* and *Salmonella* need to evaluate. And mechanism responsible for the bactericidal and anti-inflammatory effects of MccJ25 needs to be studied, and systematic scientific evaluation of MccJ25 in clinical and industrial applications needs to be addressed. In particular, convincing evidence is needed to prove the pleiotropic functions of MccJ25 not only in the eradication of clinically source MDR pathogens from the gastrointestinal tract but also in terms of maintaining homeostasis and alleviating problematic inflammatory reactions. The aim of this study was to systematically evaluate the biomedical properties, prophylactic/therapeutic approaches of MccJ25. In this paper, MccJ25 was used to evaluate its antibacterial activity against pathogens, including standard strains, clinical MDR strains, its antibacterial mechanism and stability, cytotoxic activity against cultured mammalian cells, and mutagenesis in microbial communities as a way to explore possible applications in real-world environments. In addition, the anti-inflammatory activity of MccJ25 and its potential mode of action against *E. coli* O111:B4 LPS were analyzed in this paper, and the protective activity of the MccJ25 against clinically source MDR *E. coli* infection was studied in a BALB/c mouse model.

## Materials and Methods

### Preparation of MccJ25

Microcin J25 was obtained in our lab based on previously described methods with minor modifications (24). In short, pMJ25, the specific design of the recombinant expression vector, was reworked based on the standard requirements. The recombinant vector was replicated onto an adapted *E. coli* BL21, and then the *E. coli* BL21 with the recombinant vector was allowed to grow and develop in 200 mL of medium containing kanamycin and ampicillin. Cultures were shake at 200 rpm for 16 h at 37°C. Then, the culture was centrifuged at 4°C and 12,000 rpm for 20 min to obtain raw MccJ25. MccJ25 was further purified by reverse high-performance liquid chromatography to achieve 99.95% purity MccJ25. Lyophilized MccJ25 powder was dissolved in nonendotoxemic Milli-Q water and stored in a refrigerator at -80°C until use. The amino acid sequence of MccJ25 (GGAGHVPEYFVGIGTPISFYG) in this study tested through automated Edman degradation (model 494 Procise Protein/Peptide Sequencer; Applied Biosystems, Foster City, CA) and a mass spectrometer (Q-TOF Mass Analyzer; Micromass Ltd., Manchester, UK), and the relative molecular mass (2,107.01 Da), measured by matrix-assisted laser desorption/ionization time-of-fight mass spectrophotometry with a Voyager instrument (Applied Biosystems) were consistent with the natural MccJ25 produced by the indicated bacteria.

### Standard and Clinical Strains

Strains such as *E. coli* ATCC25922, *E. coli* K88, *E. coli* K99, *E. coli* 987P, *E. coli* 1499, *E. coli* CVCC1515, *E. coli* CVCC1522, *E. coli* CVCC1543, *Salmonella enterica* CMCC50336, and *Shigella flexneri* CMCC51571 were obtained from the China Veterinary Drug Inspection Institute, Beijing. The clinical source strain LKFZ was provided by the Key Laboratory of Biological Feed Additives, Beijing. Clinically relevant lincomycin- and tilmicosin-resistant strains, *Salmonella* and *E. coli*, were provided by the College of Veterinary Medicine, China Agricultural University.

For the protective ability of MccJ25 against cocktail of clinically isolated MDR *E. coli* strains infection experiment in a mouse model, named 521ZQE, AZ1, and WWE921A-1, were ciprofloxacin-resistant *E. coli*, enrofloxacin-resistant *E. coli*, and colistin sulfate-resistant *E. coli* strains isolated from intestine of dead pigs in the present study. These indicated pathogens were kindly provided by National Veterinary Drug Evaluation Center and kept in our lab. These pathogenic microorganisms were cultured and kept in Luria-Bertani (LB) broth.

### Antimicrobial Activity Assay of MccJ25

The minimum inhibitory concentration (MIC) refers to the lowest concentration that can produce a significant hindrance to bacterial growth in a drug sensitivity test by adopting a broth dilution, with certain refinements and modifications based on the previous description (25, 26). In short, MccJ25 was dissolved in sterilized distilled water (SDW), serial 2-fold dilutions were prepared in Mueller-Hinton broth (MHB) through a 96-well microtiter plate, and the final concentration of MccJ25 was maintained in the range of 0.003125 to 256 μg/mL. Then, 2 μL of standard and clinically relevant overnight broth cultures of bacteria were inoculated into each well at a concentration of 5 × 10^6^ CFU/mL. Microtiter plates were incubated at 37°C for one full day. Two groups were set up: positive (medium with inoculum) and negative (medium only) controls. The minimum bactericidal concentration (MBC) was measured in LB medium and refers to the first drug dilution value at which the initial bacterial concentration was reduced by 99.99%. The final analysis was determined by the results of three replicated and independent experiments. To further assess and clarify the antimicrobial activity of MccJ25, the protective ability against *E. coli* adhesion, live/dead assay, and killing curve were performed, the detailed methods were provided in **Supplementary Materials**.

### Assessment of Stability and Antimicrobial Activity in Different Biochemical Conditions and Biological Fluids

In addition, we further addressed the antimicrobial activity of MccJ25. Several environmental levels were set, such as incubation at various temperature levels for 20 min and the indicated pH levels for a treatment duration of 2 h. The inhibition activity of MccJ25 under different environmental factors was measured and analyzed. The assay methods used are described above (26), with minor modifications. LB agar inoculated with *E. coli* K88 cells (approximately 10^5^ live cells per mL present). These solutions containing surviving cells are poured on plates configured with Oxford cups to form wells. Then, 150 μL of MccJ25 (1 × MIC) from the different groups previously treated with different environmental conditions was added to the well. The agar material covering the wells was treated with an overnight incubation at 37°C, and finally, the size of the diameter of the growth inhibition zone was determined.

The resistance of MccJ25 to pepsin, trypsin and chymotrypsin was evaluated based on previous studies (26), with minor modifications. *E. coli* K88 cells were prepared (approximately 1 × 10^7^ CFU/mL), and 1 mL of the bacterial suspension and MccJ25 were transferred into 9 mL of medium. For the control, MccJ25 was not added. LB agar inoculated with *E. coli* K88 cells (~10^5^ viable cells/mL) was poured onto a plate containing an Oxford cup to form individual wells. Then, 2 and 4 h MccJ25 (1 × MIC) previously subjected to different treatments were added to the wells. The agar overlay was incubated overnight at 37°C, and the diameter of the growth inhibition zone was measured.

The simulated gastric fluid (SGF) and simulated intestinal fluid (SIF) were mainly based on the relevant contents of the Chinese Pharmacopoeia as described previously (26). SGF was configurated with 10 mg/mL pepsin, 0.03 M NaCl was used to blend the pH to 1.5, while SIF was configured with 10 mg/mL trypsin, 0.05 M KH2PO4 was used to blended pH as 7. SGF and SIF were then measured separately, and the antimicrobial activity of MccJ25 in serum was determined. *E. coli* K88 cells were grown in MHB (Difco Laboratories, Detroit, MI, USA). In short, 1 mL of the bacterial suspension and MccJ25 were transferred into 9 mL of medium. The control group was as follows: no MccJ25 was added to it. *E. coli* K88 cells with a live cell count maintained at approximately 10^5^ cells per milliliter were inoculated on LB agar, which was then poured into plates equipped with Oxford cups, thus forming wells. Then, MccJ25 (1 × MIC) previously treated with SGF, SIF and serum for 2, 4, and 6 h was added to these wells. One of the agar substances covering the wells needs to be incubated overnight at 37°C, and finally, the size of its growth inhibition area, i.e., the diameter, needs to be determined.

### Preliminary Study of the Mode of Action of MccJ25 Against ETEC-Sensitive Strains

Scanning electron microscope (SEM) was performed to test the morphological features of *E. coli* K88 cells by comparing cells incubated in the absence (control) or presence of MccJ25. For SEM sample preparation, the standard protocol was used based on our previous report (26) with slight modifications. Overnight *E. coli* K88 cultures were inoculated and grown to the mid-logarithmic phase in the fresh LB medium at 37°C for 2 h, then harvested by centrifugation, washed twice with PBS and re-suspended in the same buffer. Approximately 2 × 10^7^ cells were incubated with 1 and 2 × MIC MccJ25 at 37°C for 1 h. Thereafter, cells were pooled and centrifuged at 5000 g for 5 min. Bacterial pellets were washed 3 times with PBS and centrifuged after each wash. Cells were fixed with 2.5% glutaraldehyde at 4°C overnight and washed 2 times with PBS. They were then harvested, post-fixed with 1% osmium tetroxide for 1 h and dehydrated for 15 min each in a graded ethanol series (50, 70, 80, 90, 95 and 100%). Cells were then treated with absolute acetone for 20 min, a mixture of alcohol and iso-amyl acetate (1:1) for 30 min and pure iso-amyl acetate for 1 h. Lastly, samples were dehydrated in a critical point dryer (Model HCP-2, Hitachi, Chiyoda-ku, Tokyo, Japan) with liquid CO_2_. The dehydrated samples were coated with gold-palladium and observed by SEM (Model EVO MA10 XVP, Carl Zeiss, Jena, GmbH, Germany).

Transmission electron microscopy (TEM) was conducted based on our previous study with minor modifications (26). Accurate evaluation of the morphological characteristics of *E. coli* K88 cells was achieved by comparing the morphology of cells without the addition of MccJ25 (control) and with the addition of MccJ25 (treatment group). For TEM sample preparation, the *E. coli* K88 cells were maintained at 37°C incubation temperature and allowed to develop to the mid-log phase of the growth stage before being harvested by centrifugation, washed twice with sterilized physiological saline and resuspended in the same buffer. Approximately 2 × 10^7^ cells and MccJ25 (1 and 4 × MIC) were combined and incubated for 60 min at 37°C. The cells were then aggregated together and centrifuged at 5,000 g for 5 min. The bacterial pellet was washed three times with PBS and centrifuged after each wash. Cells were fixed with 2.5% glutaraldehyde at 4°C overnight and washed twice more with PBS. Cells were then collected, postfixed with 1% osmium tetroxide for 1 h and dehydrated in a graded ethanol series for 15 minutes (50, 70, 80, 90, 95, and 100%). The cells were then treated with absolute acetone for 20 min, and the samples were transferred to a mixture of absolute acetone and resin (1:3 and 1:1) for 1 to 3 h, followed by an overnight stay in resin. Finally, the samples were placed in capsules containing embedding medium and heated at 70°C for approximately 9 h. Samples were stained with uranyl acetate and basic lead citrate for 15 min and observed by TEM (JEM-1230 type, JEOL, Tokyo, Japan).

Based on the previously worked out method (27), briefly, gel retardation experiments were carried out, in which 400 ng of plasmid DNA (pGEM-bGAL; Promega) was increasingly bound to 20 mL of binding buffer in MccJ25 binding and incubated for 1 h at room temperature. Then, 4 mL of local loading buffer in each sample was skybound, and a 12 μL aliquot was bound to a 1% agarose gel. Finally, 0.5× Tris-borate-EDTA buffer was used for electrophoresis.

*E. coli* K88 cells were incubated at 37°C until growth reached the mid-log phase stage, and the cells were washed and resuspended in 10 mM NaH2PO4, pH 7.2 (2 × 10^7^/mL). Cells were then incubated with 1 M Sytox Green for 15 min in a light-free environment. After the addition of MccJ25 (0.5 ×, 1 ×, 2 ×, 3 ×, 4×, and 5 × MIC) again, the monitoring showed an increasing fluorescence caused by the binding of the cationic dye to the intracellular DNA with increasing time (excitation wavelength of 485 nm and emission wavelength of 520 nm).

### Cells Culture

Certain adjustments were made to Dulbecco’s modified Eagle’s medium (DMEM), i.e., 20% fetal bovine serum (FBS) was added, and human colon cancer cell line Caco-2 and murine cell line macrophage RAW264.7 cells were inoculated into the adjusted medium, with the inoculum density maintained at 2 × 10^4^ cells/mL, placed in 96-well plates until the overall confluence ratio reached 80% to 90%, respectively.

### Cytotoxicity and Hemolysis Assays

To test whether MccJ25 could have an effect on the metabolic activity of the cells, the necessary MTT assay was performed (Roche Molecular Biochemistry, Mannheim, Germany) as previously described (28, 29). The whole operation process can be briefly summarized as follows: first, various concentration gradients of MccJ25 solution were added, the ambient temperature of the cell secondary incubation was maintained at 37°C, the CO_2_ concentration was maintained at 5%, and the incubation time needed to reach 24 h. The control group was the cells without any treatment. Afterwards, 20 μL of MTT solution (concentration 5 mg/mL, medium DPBS) was added to all wells and incubated for 3.5 h at a temperature of 37°C and a CO_2_ concentration of 5%. Components of the medium were removed by centrifugation, and the MTT metabolites were resuspended in 200 μL of dimethyl sulfoxide. The absorbance value at 560 nm was measured, and the background value was the absorbance at 670 nm, which needed to be subtracted from the background value to obtain the absorbance value. This absorbance value was then normalized by combining the absorbance values of 1% Triton X-100 treatment (100% cell death) and no treatment (0% cell death, control)

The secretion of the cell membrane enzyme lactate dehydrogenase (LDH) was measured on the basis of a colorimetric assay. Caco-2 and RAW264.7 cells (2 × 10^4^ cells/well) as controls without or with concentrations of 2 to 512 μg/mL MccJ25 were incubated for 24 h at 37°C in 5% CO_2_. A simple step was to transfer 100 μL of cell culture medium from each well to a new 96-well plate and measure the activity of LDH based on the LDH cytotoxicity assay kit (Clontech, Mountain View, CA, USA) according to the manufacturer’s instructions. The catalyst and dye solutions were mixed well and added to the wells containing cell culture medium and then incubated for 30 min at room temperature. The absorbance at 490 and 670 nm was measured with a microplate reader (Model SynergyMx, BioTek, Winooski, VT, USA). Absorbance values were corrected by subtracting the absorbance value at 490 nm from the absorbance value at 670 nm. Absorbance values were normalized to absorbance values from 1% Triton X-100 treatment (100% cell death) and no treatment (0% cell death, control). DMEM containing 1% Triton X-100 and 10% FBS was used as a positive control.

Fresh mouse/pig erythrocytes (mRBCs/pRBCs) were collected from well-grown animals, centrifuged at 4,200 rpm and washed well with phosphate-buffered saline (PBS) to ensure that their supernatants were free of impurities and remained clear. The liquid was added to the 96-well plate after 2-fold dilution of MccJ25 prepared with PBS, and the final concentration of RBCs was introduced at 8%. The plate was stirred less vigorously to homogenize the liquid and incubated at 37°C for 1 h. The supernatant was then centrifuged at 4,500 rpm for 10 min, and its absorbance value at 414 nm was measured. Three replicate groups were set up for all the above experiments, and the percentage hemolysis was calculated as follows: percentage hemolysis = [(A414 in peptide solution - A414 in PBS)/(A414 in 0.1% Triton X-100 - A414 in PBS)]. ×100, where 100% hemolysis refers to the absorbance of RBCs exposed to 1% Triton X-100 in the environment and 0% hemolysis refers to the absorbance of RBCs exposed to PBS. The positive control used in this experiment was melittin.

### Lipopolysaccharide (LPS) Neutralization

The neutralizing impacts of MccJ25 on LPS were assessed according to a color-emitting limestone cell lysate assay. Levels of *E. coli* LPS (1 EU/mL) were prepared and combined with various concentrations of MccJ25 (1 to 128 µg/mL) and inoculated in the wells of sterile microtiter plates at 37°C. Then, 50 μL of the above mixture was extracted, the same volume of limestone cell lysate reagent was added, and the mixture was incubated for 10 min at 37°C. A yellow color was revealed upon the addition of 100 μL of the chromogenic substrate solution. The reaction was finished by adding 25% acetic acid, and finally, its absorbance value at 405 nm was measured.

### Determination of the Mutation Rate, Resistance Acquisition and MIC Variability

Mutation rates were based on rifampicin and refined (28, 29). Overnight cultures of *E. coli* K88 were diluted in 50 mL of LB broth at a ratio of 1:10,000 and grown for 3.5 h at 37°C. The culture was diluted with LB broth without antibiotics (negative control), ampicillin (4 μg/mL, 0.25 × MIC or 8 μg/mL, 0.5 × MIC), or MccJ25 (0.25 × or 0.5 × MIC) at a 1:3 ratio. Eleven 1-mL replicates of each treatment group were grown at 37°C for 24 h. These cultures were then serially diluted and plated on LB agar plates containing 100 μg/mL rifampicin. Afterwards, plates were incubated at 37°C for 48 h. Single colonies on the plates were counted to determine CFU/mL. Mutagenesis rates were calculated using the bz-rates-mutation rate calculator (http://www.lcqb.upmc.fr/bzrates).

To clarify whether sublethal concentrations of MccJ25 would cause an increase in MIC, *E. coli* K88 cells were incubated in 1 mL of MHB containing 0.25×, 0.5×, 1×, 2× and 4× MIC in this experiment based on our previous study (29). To quantify the production status of the bacteria, the bacterial density was measured after incubating for 24 h. Cultures with the second highest concentration allowed to grow (OD600 ≥ 2) were diluted 1:100 with MccJ25 MHB at different concentrations than those described above. Cultures that grew above the indicated MIC levels were plated on LB agar plates, and their MIC levels were determined by the broth macrodilution method. Ampicillin, tetracycline, and ciprofloxacin at 0.25×, 0.5×, 1×, 2× and 4× MIC, respectively, were applied as controls, and sequential passages were performed daily for a period of 25 days.

To measure the degree of MIC variability, *E. coli* K88 cells were cultured for 5 days in untreated MHB, MHB containing ampicillin (0.25 × MIC, positive control), or MHB containing MccJ25 (0.5 × MIC). A 1:100 dilution of the bacteria was performed daily in the above MHB. Aliquots of the culture medium were used daily for 5 days thereafter to determine the MICs of ampicillin, tetracycline, aureomycin, and kanamycin. This study was performed based on our previous study (29).

### Mice Model

This study including two infected mice experiments was conducted mainly following the provisions in the Chinese Code of Welfare and Ethics for Laboratory Animals. This experimental protocol was approved by the Institutional Animal Care and Use Committee of China Agricultural University (CAU No. AW04101202-1-1) and confirmed by the Regulations for the Administration of Affairs Concerning Experimental Animals of the State Council of the People’s Republic of China [No. SYXK(Jing) 2015-0028].

### LPS-Challenged Mice Experiment

Female BALB/c mice (5-6 weeks) was provided by HFK Bioscience Co. (Beijing, China). All mice were raised in laminar flow cabinets and housed individually in a room with strictly controlled temperature and humidity, where a 12-hour light and 12-hour dark cycle was implemented, and mice had free access to food and water throughout the experimental period.

*E. coli* O111:B4 LPS (Sigma-Aldrich) at a concentration of 15 mg/L was injected into the peritoneal cavity of female BALB/c mice (age 7 weeks, weight 15 ± 0.5 g), and approximately 30 min after the challenge of *E. coli* O111:B4 LPS, MccJ25 was orally gavaged to the mice at a concentration of 4.55 or 9.1 mg per kg body weight (BW). The concentrations of MccJ25 were selected based on our previous study (24). Mice were followed for 72 h to test the survival rate of mice that were later sacrificed. However, for LPS treated mice, when the lifespan of the mice was around 50%, the mice were sacrificed. The serum from mice were obtained. The lung, liver, spleen, jejunum, and ileum were harvested, kept in liquid nitrogen immediately, stored at -80°C until analysis. For histopathological assessment, tissues and organs were fixed using 4% paraformaldehyde, hematoxylin eosin (H&E) was applied to examine the histological and pathological changes according to the standard protocol as described previously (24).

### Mouse RAW264.7 Cells Model

The mouse macrophage cell line RAW 264.7 was obtained in our lab and stored in liquid nitrogen. Cells were cultured in DMEM supplemented with 1% antibiotics (100 U/mL penicillin and 100 g/mL streptomycin) with 20% FBS at 37°C and 5% CO_2_. Cells were transferred and inoculated in 12-well plates (1 × 10^4^ cells/well) and cultured for 24 h.

RAW 264.7 cells (approximately 2 × 10^4^ cells/well) were cultured with 1 µg/mL *E. coli* LPS for 3 h at 37°C in the absence (control) or presence of 1 μg/mL MccJ25 at 5% CO_2_. The cells and cell culture supernatants were then collected. Culture supernatant mixed with Griess reagent (1:1) and incubated for 15 min at room temperature. A microplate reader was used to measure nitric oxidase (NO) at 540 nm. The changes in induced NO levels were clarified based on the comparison between the values of the treated and control groups. Each experiment was repeated three times.

### Protective Capacity of MccJ25 Pretreatment on Cocktail of MDR *E. coli S*trains Challenge in Mice

4-week-old C57BL/6 J female mice [initial body weight (BW) 15.23 ± 0.09] were purchased from Charles River Laboratories (Beijing, China) and raised under standard conditions. The producer was described in “Mice Model” section. Examination of previous excrement shaping on MacConkey agar (Beijing Aoboxin Biotechnology Co., Ltd., Beijing, China) showed that no elevating enterobacter or *E. coli* were found in the analyzed mice as previously described (24). Mice were maintained in a 3-d acclimatization period of time. Afterwards, 36 mice were randomly separated into three experimental groups based on BW: Uninfected group, control; Infected group; MccJ25+infected group, 12 mice per group in four cages, three mice/cage, total of 12 cages. Cocktail of MDR *E. coli* strains infection producers were performed as our previous described previously (30), experimental roadmap was shown in **Figure 10A**. A single of colony of MDR *E. coli* strains 521ZQE, AZ1, and WWE921A-2 were incubated in LB medium and grew to stationary stage (OD600 > 3), respectively. All mice were pretreated 0.2 ml PBS (control and infected group) or 0.2 ml PBS containing with 9.1 mg/kg/BW MccJ25 (MccJ25+infected group) for 3 d (once/day) prior to infection. Infected mice and MccJ25+infected mice received 0.2 ml sterile PBS containing 5 × 10^8^ cocktail of MDR *E. coli* strains by oral gavage. The control group still received 0.2 ml of sterile PBS by gavage for 2 d. The concentration of biosynthetic MccJ25 was selected based on our previous study (24). After 3 d, mice were euthanized friendly, intestinal tissues, organs and serum were obtained. Fresh fecal, tissues, liver, and spleen were immediately placed on ice (1-2 h) and transported to the laboratory to count the specific bacteria in the laboratory. Serum was kept in -20°C and part of jejunum, colon, and spleen were stored in the -80°C for cytokines analysis.

### Clinical Signs Determination and Bacterial Load Counting

In the LPS-challenged mice experiment, lifespan of mice was monitored for 72 h. In the cocktail of MDR *E. coli* strains infection experiment, BW change, rectal temperature, diarrhea scores and fecal image, or survival rate were measured. For the diarrhea score evaluation producer was referred to previous study (24).

The 0.1 g fresh jejunum, ileum, proximal colon, feces, liver, and spleen were collected and kept in sterile tubes containing 0.9 ml sterile physiological saline solution. Then, cryogenic lapping instrument (Shanghai Jingxin Industrial Development Co., Ltd., China) was applied to prepare the homogenate. 0.1 mL indicated samples prepared by ten-fold serial dilutions method were plated on indicated antibiotics MacConkey (OXOID, Hampshire, UK) and ChromAgar™ (OXOID, Hampshire, UK) plates. These plates were cultured overnight at 37°C to count colonies. Colony numbers were expressed as log 10 CFU/g of samples. All counts were performed in triplicate. Colonies were observed on the MacConkey agar and ChromAgar plates in the absence of indicated antibiotics, we considered that they were enterobacteria-like bacterium belonging to the class of family Enterobacteriaceae.

### Quantification of Cecal Specific Bacteria

Quantification of total bacteria, *E. coli, Lactobacillus*, and *Bifidobacterium* was done using PCR. In brief, total microbial DNA from cecal digesta was obtained. The total bacteria, *Lactobacillus*, *Bifidobacterium*, *E. coli* primers were synthesized in Invitrogen (Shanghai, China), which are listed in **Table S1**. Standard curves of indicated bacteria were generated based on the construction of standard plasmids containing the 16S rRNA genes. The copy numbers of total bacteria, *Lactobacillus*, *Bifidobacterium*, and *E. coli* were calculated referring to standard curves as described previously (30).

### ELISA Analysis

The concentrations of tumor necrosis factor-α (TNF-α), interleukin-6 (IL-6), IL-4, interferon (IFN)-γ, and nitric oxide (NO) in serum were measured. In addition, TNF-α and NO levels were also measured in the supernatant of RAW264.7 cells. ELISA was performed using a commercial ELISA kit (eBioscience, San Diego, USA). LPS levels in plasma were analyzed using the QCL-1000 kit (Xiamen, China) based on the manufacturer’s standards. On the day of sacrifice, eyes were extracted with ethylenediaminetetraacetic acid (EDTA), and blood was collected into clean test tubes. Peripheral hemograms and white blood cells (WBCs) were analyzed using a Coulter LH755 hematology analyzer. The levels of serum D-lactate were measured using ELISA kit (Jiancheng, Nanjing, China) according to the manufacturer’s protocols.

### RT–PCR Analysis

The extent and mRNA expression levels of *TNF-α, IL-6, IL-10, IL-1β*, IFN*-γ*, and *TLR4*, and *NF-κB* in each body part of the jejunum, ileum, colon, and spleen and in RAW264.7 cells were clarified by RT–PCR as previously described (31). Primers for *TNF-α, IL-6, IL-10, IL-1β, IFN-γ*, *TLR4*, and *NF-κB* are described in **Table S1**. The specific assay steps were as follows: total RNA was extracted from the jejunum, ileum, spleen, and cells by TRIzol reagent (Invitrogen, Carlsbad, CA). Combined with gel electrophoresis and a NanoDrop 2000 spectrophotometer (Thermo Fisher Scientific, Wilmington, DE), the quality and quantity of total RNA were determined. First-strand cDNA was synthesized from the collected RNA (1 μg) according to the description of the Prim-Script First-Strand cDNA Synthesis Kit (Takara, Otsu, Japan), and the results indicated that the kit is suitable for real-time PCR normalization of the relative amounts of mRNA to GAPDH processing. Then the 2^-ΔΔCt^ method was used for the next step of analysis.

### Western Blotting Analysis

Frozen tissue samples and fresh cells were homogenized in RIPA lysis buffer containing protease inhibitors (Applygen, Beijing, China). Protein levels were determined using a BCA protein assay kit (Thermo Fisher Scientific, Rockford, IL). Thirty micrograms of tissue and cell protein samples were electrophoresed on SDS polyacrylamide gels and electrotransferred to PVDF membranes (Millipore). The membranes were blocked with 1 × TBST (Sigma–Aldrich, St Louis, MO) containing 5% skim milk or bovine serum albumin (Sigma-Aldrich, St Louis, MO) for 1 h at room temperature, and then the membranes were treated with the corresponding TLR4, myeloid differentiation factor 88 (MyD88), TNF-α, IL-6, phosphorylated p65, phosphorylated p38, and IκB (Cell Signaling Technology, Boston, MA) primary antibodies (1:1000 dilution) overnight at 4°C. Afterwards, the membrane was washed with 1 × TBST 5 times for 5 min each time, and the membranes were incubated with horseradish peroxidase-conjugated goat anti-mouse IgG (Huaxing Biotechnology, Beijing, China) for 1 h at room temperature. GAPDH and β-actin were used as homekeeping gene (Cell Signaling Technology, Boston, MA). Chemofluorescence was detected using the ImageQuant LAS 4000 mini system (GE Healthcare Biosciences AB, Inc., Sweden) with Western Blot Luminance Reagent (Applygen, Beijing, China), and the membranes were cleaned with an ImageQuant TL (GE Healthcare Life Science) gel imaging system for quantification.

### Statistical Analysis

Experimental results are presented as the mean plus mean standard error (SEM). One-way ANOVA was performed on the data in Prism 8 software (GraphPad Software Inc., San Diego, CA, USA). The survival determination of mice was performed using Graph-Pad Prism 8 (GraphPad Software, La Jolla, CA). Statistical difference test of survival curves was analyzed by the Mantel-Cox (log rank) Tukey’s *post-hoc* test was used to clarify the level of significance of differences between treatments for other results. The nonparametric Mann-Whitney test was used to look for differences in fecal shedding and organ colonization between the infected and MccJ25 groups. The Chi-Square test (likelihood ratio) was performed to examine the frequency of positive animals in various *E. coli* levels in intestinal segments. All data were visualized using Prism 8. A *P* value < 0.05 indicates that the differences are statistically significant. Unless otherwise stated, all experiments were performed three times independently.

## Results

### Antimicrobial Activity Assay

The antimicrobial activity of the indicated concentrations (from 0.0125 μg/mL to 512 μg/mL) of MccJ25 against standard strains and clinical source drug-resistant pathogens is listed in **Tables 1**, **2**, respectively. Gradient dilutions of purified MccJ25 were cultured with the indicated standard strains (**Table 1**) and clinically relevant veterinary drug-resistant bacteria (**Table 2**), and then the MICs were determined. MccJ25 was highly effective and had a low MIC/MBC against all standard strains and clinically relevant gram-negative antibiotic-resistant microorganisms tested. Furthermore, for standard strains, the lowest MIC value of MccJ25 against the highly sensitive strains was 0.03 μg/mL, including *E. coli* K99, *E. coli* 987P, *S. pullorum* CVCC1791 and two ETEC strains (*E. coli* CVCC1522 *and* CVCC1543). For clinically relevant veterinary MDR the lowest MIC value of MccJ25 against the highly sensitive strains was 0.03 μg/mL, including one *E. coli* strain and four *Salmonella* strains.

**Table 1:** MIC and MBC comparison of antimicrobial peptide MccJ25 among standard pathogens.

Strains	MIC^a^, μg/mL	MBC^b^, μg/mL	MIC/MBC
**Gram-negative bacteria**			
*E. coli* ATCC25922	0.125	0.500	0.250
*E. coli* K88	0.250	0.500	0.500
*E. coli* K99	0.030	0.250	0.120
*E. coli* 987P	0.030	0.500	0.060
*E. coli* 1499	0.400	1.000	0.400
*E. coli* CVCC1515	0.500	2.000	0.250
*E. coli* CVCC1522	0.030	0.500	0.060
*E. coli* CVCC1543	0.030	0.250	0.120
*Salmonella enteritidis *CMCC50336	2.000	4.000	0.500
*Shigella flexneri *CMCC51571	0.125	0.500	0.150

^a, b^All MIC and MBC values represent three independent experiments.

**Table 2:** MIC and MBC of antimicrobial peptide MccJ25 among clinical isolates.

Strains	Source^a^	MIC^b^, μg/mL	MBC^c^, μg/mL	MIC/MBC
***E. coli***				
SWI 437	Pig stool	0.030	0.250	0.120
SWI 441	Pig stool	0.060	0.500	0.120
SWI 541	Pig stool	0.500	2.000	0.250
SWI 535	Pig tissue	0.500	2.000	0.250
SWI 531	Pig tissue	0.060	0.500	0.120
LKFZ GZM4	Pig stool	0.250	1.000	0.250
LKFZ GL2	Pig stool	0.250	0.500	0.500
LKFZ F2	Pig stool	0.250	0.500	0.500
BRO 134	Broiler stool	0.250	2.000	0.125
BRO 528	Broiler stool	0.500	2.000	0.250
BRO 477	Broiler stool	0.500	1.000	0.500
CATT921A-1	Mastitis pathogens	0.250	2.000	0.125
521ZQE	Pig tissue	0.250	2.000	0.500
WWE921A-2	Pig tissue	1.000	2.000	0.500
AZ1	Pig tissue	0.500	1.000	0.500
***Salmonella***				
CATT W14	Mastitis pathogens	0.060	0.250	0.240
CATT LX9	Mastitis pathogens	0.060	0.125	0.480
CATT LX5	Mastitis pathogens	0.030	0.125	0.240
CATT LX2	Mastitis pathogens	0.030	0.125	0.240
CATT LX1	Mastitis pathogens	0.060	0.250	0.240

^a^Strains named LKFZ were provided by Beijing Bio-feed Additive Key Laboratory. Clinical source strains designated SWI, BRO, CATT were provided by Dr. Congming Wu, College of Veterinary Medicine, China Agricultural University). These clinical source strains, SWI, BRO and CATT were antibiotic (Colistin sulfate, lincomycin and tilmicosin) resistant Salmonella and E. coli. E. coli 521ZQE, WWE921A-2, and AZ1 were clinically isolated from intestine of dead pig multidrug-resistant strains (Colistin sulfate, enrofloxacin, ciprofloxacin). ^b,c^All MIC and MBC values represent three independent experiments.

### Microcin J25 Kills Enterotoxigenic *E. coli* K88

Enterotoxigenic *E. coli* (ETEC) is a pivotal pathogenic bacterium that induces diarrhea incidence in newly/postweaned pigs and is also linked to diarrhea disease in infants, young children, and travelers (30–32).

**Figure 1:**
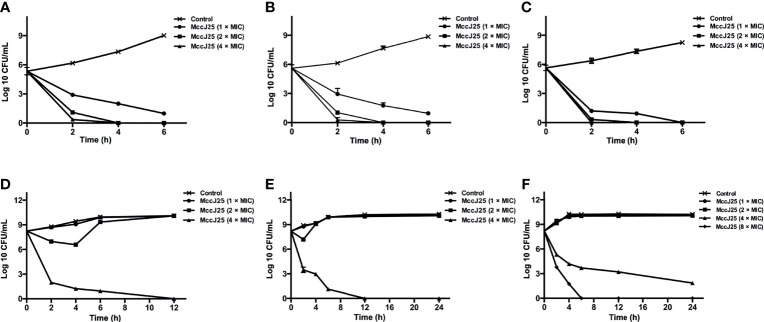
Bactericidal activity assay of MccJ25 against *E coli* K88. Different concentrations of *E coli* K88 were cultured in LB containing different levels of MccJ25. *E coli* K88 cells (5 × 10^5^ CFU/mL) were incubated and grown to different stages, including early-log **(A)**, late-log **(B)** and stationary **(C)** phases. Additionally, bacteria, 5 × 10^8^ CFU/mL, were cultured and grown to early-log **(D)**, late-log **(E)** and stationary **(F)** phases. Data are means ± standard error of mean from 6 biological replicates.

Therefore, we examined the bactericidal activity of MccJ25 against ETEC K88-sensitive strains at different growth stages (i.e., early log phase, late log phase, and stationary phase). The results showed that MccJ25 exerted potent antimicrobial activity against ETEC K88 regardless of the growth phase (as shown in **Figure 1**). In the early log phase (**Figure 1A**), late log phase (**Figure 1B**) and stationary phase (**Figure 1C**), MccJ25 was shown to inhibit the bacteria to a greater extent. Within each growth period, cells were incubated with different concentrations of MccJ25, and all inoculums (5 × 10^5^ CFU/mL) were killed by MccJ25 (2 × MIC) within 4 h. There was also no tendency of bacterial regrow. In addition, a reduction of 5 log values was found after 2 h incubation with MccJ25 (4 × MIC), and there was no tendency for bacterial cells to regrowth during the resting period. Although the antimicrobial activity of MccJ25 at the indicated MICs was decreased, it still exerted strong antimicrobial activity against ETEC K88, resulting in an approximately 4-log reduction at 2 h during all phases. Furthermore, all inoculants were killed within 6 h by MccJ25 at the indicated MICs during the stationary phase.

To test if MccJ25 was able to be applied to treat disease cause by pathogenic microorganisms in late stage of infection, we imitated the late stage of bacterial infection using a large amount of bacteria (5 × 10^8^ CFU/mL). When 5 × 10^8^ CFU/mL bacterial cells at the log phase were incubated with MccJ25 (4 × MIC), after incubation for 12 h, an 8-log reduction was observed. No significant difference was observed in susceptibility to different concentrations of MccJ25, but a 6-log reduction was observed within 7 h of MccJ25 (1 × MIC) treatment. MccJ25 killed ETEC K88 efficiently, regardless of the bacterial growth phase (**Figures 1D–F**). Additionally, adhesion assays (**Figure S1**) and Live/dead assay (**Figure S2**) were performed to further investigate the strong bactericidal activity of MccJ25. The results may suggest that MccJ25 is a potent candidate for killing ETEC K88 by damaging the bacterial cell wall and membranes.

MccJ25 was highly effective and had a low MIC/MBC against all standard strains and clinically relevant gram-negative antibiotic-resistant microorganisms tested. For standard and clinically relevant veterinary drug-resistant bacterial strains, the MIC (0.03 μg/mL) and MBC (0.25 μg/mL) values of MccJ25 against the highly sensitive strains were the lowest. These findings demonstrated that MccJ25 had prominent bactericidal activity against *E. coli* pathogens, especially killing antimicrobial-resistant pathogens, providing potential perceptiveness as a traditional antibiotic alternative treatment method.

### Potential Mode of Action of MccJ25 Against the Sensitive Strain ETEC

**Figure 2:**
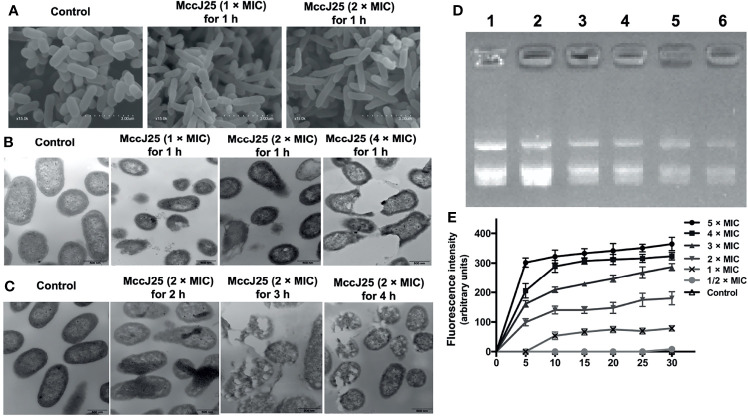
Potential mode of action of MccJ25. **(A)** Morphological analysis of the ETEC K88 membrane with MccJ25 by scanning electron microscopy. 10^7^ CFU/mL of ETEC K88 cells were treated with different concentrations (1, 2 × MIC) MccJ25 for 1 h, scanning electron microscope was performed to test the change of morphology. **(B)** Transmission electron microscopy micrographs of ETEC K88 cells treated with different concentrations of MccJ25 for 1 h **(C)**. TEM micrographs of ETEC K88 at 2 × MIC concentrations of MccJ25 for different durations. **(D)** The interrelationship that exists between the peptide and plasmid DNA. The specific status of binding was clarified by measuring the inhibition exerted on the migration of plasmid DNA. Various amounts of MccJ25 were incubated separately with 400 ng of MccJ25 for one hour at room temperature prior to electrophoresis on a 1.0% agarose gel. The weight ratios (MccJ25:DNA) are expressed as follows: lane 1, plasmid DNA alone; lane 2, ratio 1:4; lane 3, ratio 1:2; lane 4, ratio 1:1; lane 5, ratio 2:1; lane 6, ratio 4:1. **(E)** Time dependence of Sytox Green influx into *E coli* K88 cells. Cells were incubated with 1 M Sytox Green until basal fluorescence was reached and maintained in a steady state. Then, MccJ25 was added, and the fluorescence was measured at fixed time points (excitation and emission wavelengths of 485 nm and 520 nm, respectively). Error bars indicate mean ± standard error of mean (*P <* 0.005).

Evidence has demonstrated that microcins have different mechanisms against different sensitive pathogens (16, 33–36). Based on the live/dead assay, to investigate the mechanism of action of MccJ25, cells were incubated with *E. coli* K88 at different concentrations (1 × and 2 × MIC). Cells were examined by SEM, which allowed us to directly observe their morphology after MccJ25 treatment (**Figure 2A**). After incubation of *E. coli* K88 cells with MccJ25 (1 × and 2 × MIC) for 60 min, they became filamentous and elongated, indicating that bacterial cells treated with biosynthetic MccJ25 failed to divide normally. Moreover, the membranes of biosynthetic MccJ25-treated *E. coli* K88 cells were rougher, and blebbing was observed, as evidenced by the formation of aggregates on the cell surface compared to the control, which showed a translucent and smooth surface without cellular debris.

To further investigate the mode of action of MccJ25, we investigated the possible mechanism by which MccJ25 disrupted the cytoplasmic membranes of ETEC cells. MccJ25 was incubated with ETEC K88 at different concentrations for 0.5 and 1 h, and the morphological features of bacteria were examined by TEM. As shown in **Figures 2B, C**, the integrity of ETEC K88 membranes was compromised to some extent after treatment with different concentrations of MccJ25 for different growth stages. The membranes of *E. coli* K88 cells treated with MccJ25 (1 ×, 2 × and 4 × MIC) for 0.5 and 1 h were disrupted, thereby altering the overall morphology of the bacterial cells. Translucent cytoplasmic zones were also observed. Moreover, the outer cytoplasmic surface was thicker, and blebs were evident on the cytoplasmic membranes of *E. coli* K88 cells. Additionally, ETEC K88 cells treated with MccJ25 showed evidence of cytoplasmic vacuolation and membrane invagination, reflecting a greater degree of impairment of bacterial membrane integrity after prolonged treatment.

The DNA-binding capability of MccJ25 was also examined in ETEC K88 cells (**Figure 2D**). The results of DNA binding experiments indicated that when the weight ratio of MccJ25 was kept above 2.0, the migration movement of DNA was slightly inhibited. As shown in **Figure 2E**, there was no change in intracellular Sytox fluorescence because Sytox green cannot cross intact membranes (control). This was confirmed by the increasing dose dependence of Sytox fluorescence, and membrane integrity was significantly disrupted when MccJ25 was added to ETEC K88 cells. Based on these results, we conclude that MccJ25 disrupted the cytoplasmic membranes of the ETEC-sensitive strain.

### Stability and Antibacterial Activity of MccJ25

**Figure 3:**
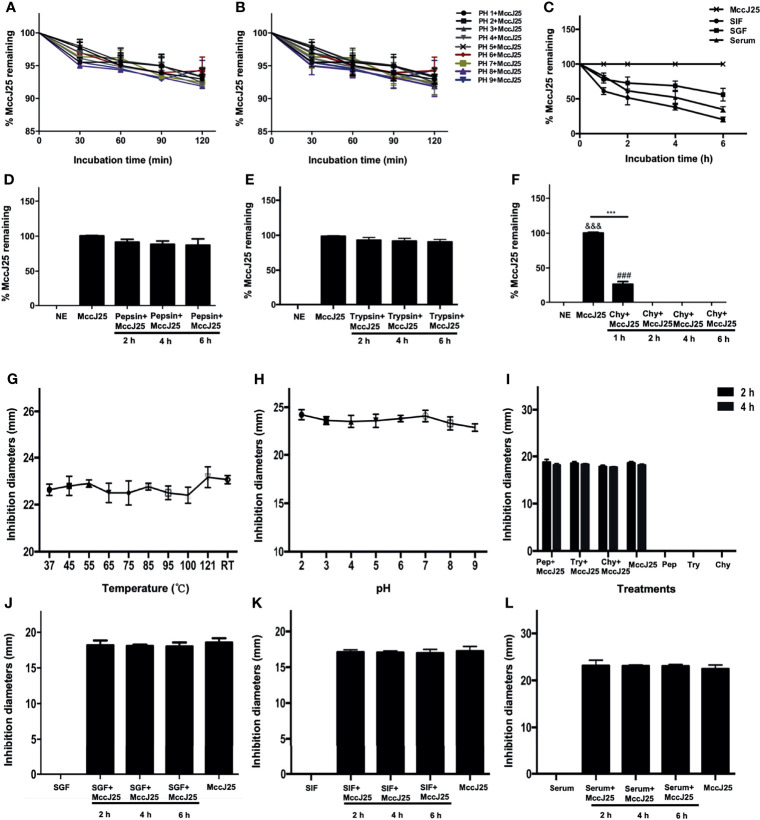
Stability and activity of MccJ25 in different conditions and biological fluids. Various concentration of MccJ25 was incubated with different temperatures **(A)**, pH values **(B)**, **(C)** SIF, SGF, and serum. Proteases **(D-F)** for different indicated times. Afterwards, the amount of the remaining MccJ25 was determined by RP-HPLC. All data in this paper are given as the mean ± standard error of mean (n = 6). ^***^ *P* ≤ 0.001 indicates MccJ25 compared to chymotrypsin plus MccJ25 (1 h); ^&&&^ *P* ≤ 0.001 indicates MccJ25 compared to chymotrypsin plus MccJ25 (2, 4, and 6 h, respectively); ^###^ *P* ≤ 0.001 means chymotrypsin plus MccJ25 (1 h) compared to chymotrypsin plus MccJ25 (2, 4, and 6 h, respectively). NE: negative control; Chy: chymotrypsin. (G-L) MccJ25 maintains its strong antibacterial activity under different conditions. Effects of temperature **(G)**, RT: room temperature, pH values **(H)**, proteases **(I)**, SGF **(J)**, SIF **(K)**, and serum **(L)** on the inhibition of *E coli* K88 by MccJ25. The zone of inhibition is often used to clarify the antimicrobial activity of MccJ25. The results showed that no significant differences were demonstrated between the treatment groups (*P* > 0.05). Chy refers to chymotrypsin. Data are presented as the mean of 6 biological replicates ± standard error of mean form.

To examine the therapeutic potential of MccJ25, we assayed its stability after exposure to different temperatures, pH values, proteases, simulated gastrointestinal fluids and serum. The RP-HPLC results showed that intact MccJ25 was able to maintain its own steady state when subjected to a high temperature of 121°C and a pH environment in the range of 2.0 to 9.0 (**Figures 3A, B**). Moreover, the concentration of intact MccJ25 decreased slowly in SGF, reaching its lowest concentration within 6 h. In contrast, the degradation of MccJ25 in SIF and serum was more rapid; its half-life was 2 h in SIF and 4 h in serum. However, intact MccJ25 was still present after 6 h of incubation in SGF, SIF and serum (**Figure 3C**). After exposure to different proteases, the presence of intact MccJ25 was evaluated. More than 70% of MccJ25 was detected after treatment with pepsin and trypsin for 6 h (**Figures 3D, E**). However, after exposure to chymotrypsin for 1 h, 70% of MccJ25 was degraded, and it was below the detection limit of HRLC after 6 h (**Figure 3F**).

### MccJ25 Kills Pathogens in Simulated Gastrointestinal Tract Conditions

Based on the aforementioned results, the bactericidal potency of MccJ25 was tested under different incubation conditions (**Figure 3**). Thermal stability experiment results showed that the temperature maintained at 121°C and maintained for 20 minutes did not affect the antibacterial ability of MccJ25. The growth inhibition zone of MccJ25 against *E. coli* K88 cells was greater than 22 mm. The results were essentially the same as those of the temperature assay, where the bactericidal activity of MccJ25 was not significantly affected by a continuous increase in pH from 2.0 to 9.0. Similarly, the growth inhibition zone of MccJ25 against *E. coli* K88 cells was greater than 22 mm (**Figures 3G, H**). Although the level of intact MccJ25 exposed to chymotrypsin for 2 and 4 h was below the detection limit, the strong antimicrobial activity of MccJ25 was retained after challenge with the indicated protease for 2 and 4 h, as shown in **Figure 3I**. MccJ25 exerted strong bactericidal activity against *E. coli* K88 cells in the presence of SIF, SGF or serum. The activity of MccJ25 treated with SIF, SGF or serum was essentially the same as that of MccJ25 subjected to a single treatment (**Figures 3J–L**). These results indicate that MccJ25, which was produced *via* a reconstructed gene cluster, is possibly stable in the gastrointestinal tract and a candidate for therapeutic use in clinical settings.

### Cytotoxicity and Hemolytic Activity of MccJ25

**Figure 4:**
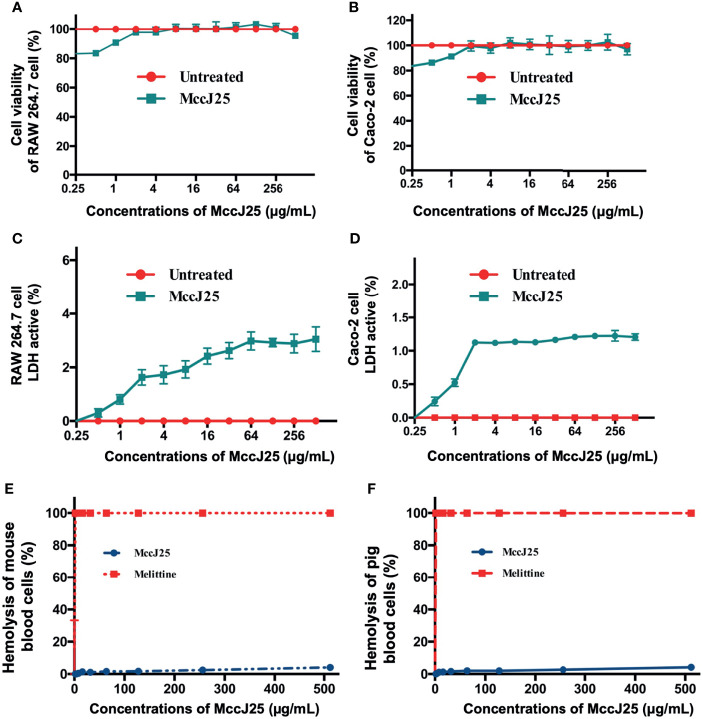
MccJ25 shows no cytotoxicity. The effects of MccJ25 on the viability of RAW264.7 **(A)** and Caco-2 **(B)** cells. The effects of MccJ25 on LDH activity in RAW264.7 **(C)** and Caco-2 **(D)** cells. Hemolytic activities of MccJ25 against mouse red blood cells **(E)** and pig red blood cells **(F)** compared with those of melittin. Error bars indicate mean ± standard error of mean. Mean values are presented from 6 biological replicates.

Even though antimicrobial agents can have an impact on pathogens, there are also host health risks. Therefore, it is necessary to enrich the form of tools for risk assessment. The first session was to determine whether MccJ25 could affect cell viability and cause cell membrane damage. MTT and LDH activity assays were performed to examine the change in cell viability of cells and LDH activity of RAW 264.7 and Caco-2 cells after treatment with various concentrations of MccJ25, respectively. After treatment of RAW 264.7 (**Figure 4A**) and Caco-2 cells (**Figure 4B**) with different concentrations of MccJ25, no significant differences in cell survival were revealed between the treated and control groups, and 1% Triton was used as a positive control and exhibited 100% cytotoxicity activity (not shown). Additionally, treatment of RAW 264.7 and Caco-2 cells with MccJ25 did not stimulate LDH activity, indicating that the integrity of the plasma membrane of RAW 264.7 (**Figure 4C**) and Caco-2 cells was maintained in the presence of MccJ25 (**Figure 4D**).

The hemolysis of MccJ25 against blood cells was determined. This was reflected by the ability of MccJ25 at serially diluted concentrations to lyse porcine and mouse erythrocytes (**Figures 4E, F**). The incubation of pRBCs and mRBCs with different concentrations of MccJ25 (0.25-512 μg/mL) resulted in minimal cell lysis at all the concentrations tested. MccJ25 at the highest concentration of 512 μg/mL incubation caused hemolysis of pRBCs and mRBCs at 4.07% and 4.16%, respectively. In addition, it has been reported that the toxin in honeybee venom possesses very potent broad-spectrum antimicrobial activity, especially at a concentration of 0.5 μg/mL, where its hemolytic activity reached 100%. Moreover, the possible development of bacterial resistance to MccJ25 when the drug is repeatedly applied several times needs to be based on the assessment of the safety of MccJ25.

### MccJ25 Does Not Induce Acquired Resistance

**Figure 5:**
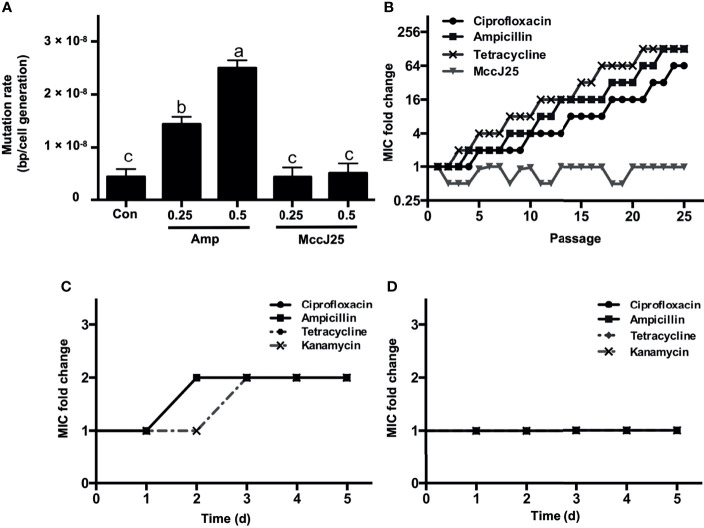
MccJ25 shows no propensity to induce resistance. **(A)** Mutagenesis rate of *E coli* K88 without drug treatment (negative control), 1 μg/mL ampicillin (0.25 × MIC), 2 μg/mL ampicillin (0.5 × MIC), 0.25 × MIC MccJ25, and 0.5 × MIC MccJ25 treatments. The results are presented by means ± standard error of mean form. Different letters indicate significant differences between means (*P* < 0.05). **(B)** Dynamics of MIC in treatments with sub-MIC concentrations of MccJ25, ciprofloxacin, ampicillin, and tetracycline after 25 repetitions of consecutive passages. The highest MIC values during each day are marked in the graph. Six biological replicates are represented in the figure. **(C, D)** Multiple resistance acquisition in *E coli* K88 exposed to sub-MIC concentrations of MccJ25 and antibiotics. The multiplicative changes in the MIC of ciprofloxacin, ampicillin, and tetracycline in an environment treated with 1 μg/mL ampicillin (0.25 × MIC) **(C)** or 0.25 × MccJ25 **(D)** for 5 days are shown. The results are presented as the means ± standard error of mean of 6 biological replicates.

Microbial acquired resistance is an important indicator for clarifying the lifetime frame of novel antibiotics, and there is a correlation between microbial mutation rates and resistance to antibiotics (37, 38). Therefore, the induction of antibiotics with lower mutation rates possesses a longer timeframe than antibiotics with higher microbial mutation rates. In this paper, we determined the magnitude of mutation rates in *E. coli* K88 cells treated with low concentrations of MccJ25 and ampicillin at sublevels of MIC. The results showed that the mutation rate of untreated *E. coli* K88 cells was approximately 5 × 10^7^ bp/cells/generation (**Figure 5A**). *E. coli* bacteria treated with 0.25 × or 0.5 × MIC ampicillin exhibited higher mutation rates (1.5 × 10^8^ bp/cells/generation and 2.5 × 10^8^ bp/cells/generation, respectively). However, *E. coli* bacteria treated with sublethal concentrations (0.25 × and 0.5 × MIC) of MccJ25 did not show a greater difference in mutation rate compared to the control, so it can be concluded that MccJ25 does not possess a sufficiently significant mutation rate to establish antibiotic resistance.

To further test whether resistance against MccJ25 increased during treatment, we induced bacteria persistently at sub-MIC levels based on the action of MccJ25. Changes in MIC after MccJ25 treatment were compared to those in the absence of antibiotic treatment (negative control) and in the presence of ampicillin (positive control). No resistant mutants were detected during the 25-d passage period (**Figure 5B**). However, sublethal levels of ciprofloxacin, ampicillin and tetracycline caused resistance in bacteria within 5, 4 and 3 days of serial passage, respectively, indicating that MccJ25 has excellent antimicrobial activity without inducing detectable resistance over a prolonged period.

We also clarified the potential ability of MccJ25 to enhance complex resistance to other types of antibiotics based on the determination of the multiplicative increase in MIC after treatment with MccJ25. After *E. coli* K88 cells were cultured under ampicillin (0.25 × MIC) imposed conditions for 5 days, the MICs of ciprofloxacin, ampicillin, tetracycline and kanamycin were all elevated to 2-fold (**Figure 5C** **),** whereas no changes were observed in the MICs of the four antibiotics in bacterial cells treated with MccJ25 (0.25 × MIC) (**Figure 5D**), indicating that it did not raise cross-resistance against other antibiotics.

### MccJ25 Prolong the Lifespan of LPS-Treated Mice

**Figure 6:**
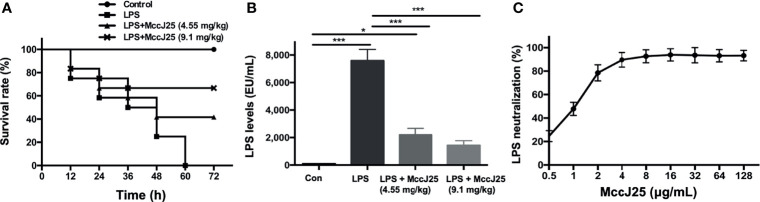
MccJ25 inhibited the LPS-induced decrease in the survival rate. **(A)** Addition of the AMP MccJ25 increased the probability of survival in mice with septic shock induced by *E coli* O111:B4 LPS (15 mg/kg/BW). **(B)** The LPS concentration in the mice plasma. **(C)** *In vitro E coli* O111:B4 LPS neutralization by the antimicrobial peptide MccJ25. Data are indicated as the means ± standard error of mean from 6 biological replicates. *P < 0.05, ***P < 0.001.

To determine and judge the therapeutic ability exhibited by MccJ25 in response to a severe sepsis model, LPS (at a dosage of 15 mg/kg body weight) was administered to BALB/c mice, and then 4.55 and 9.1 mg/kg body weight (BW) MccJ25 were injected into the mice 72 hours after treatment. As shown in **Figure 6**, all mice died after injection of LPS into mice, and the survival rate of mice exhibited a significant decrease when mice were treated with LPS for 60 h. However, the survival rate of mice treated with MccJ25 was increased, and the lifespan of mice was prolonged (**Figure 6A** **).**

Additionally, LPS levels in plasm were measured. We found that plasma LPS concentrations in the MccJ25-treated group were significantly decreased compared to those of mice treated with LPS only (**Figure 6B**). Furthermore, as described previously, limulus amebocyte lysate (LAL) is a highly sensitive indication present in glass noneutralizing LPS. To further evaluate the interaction of MccJ25 with LPS, a LAL test was performed. On the basis of this experiment, we clarified the specific function possessed by the peptide MccJ25 to neutralize LPS. The neutralization of LPS (1 EU/mL) by MccJ25 under different concentration treatments was determined in an *in vitro* environment by the lymphocyte lysate method. The results showed that MccJ25 was able to neutralize LPS and that MccJ25 neutralized LPS in a dose-dependent manner, showing 47.83% and 92.59% neutralization at 1 and 8 μg/mL, respectively (**Figure 6C**). However, the mode of action of neutralizing LPS needs to be studied in future studies.

### MccJ25 Ameliorates *E. coli* LPS O111:B4 Challenge-Induced Inflammation in BALB/c Mice

Based on the above results, MccJ25 can exert worthy properties of relevance to medical use, including strong antibacterial activity, specific mechanisms of action and high stability, and safety activity. Therefore, MccJ25 should have progressed toward clinical evaluations. It is critical to note that MccJ25 can be used for therapy in clinical settings to decrease inflammatory responses induced by pathogens or LPS. It is also able to play a role in improving inflammatory bowel disease, taking a preventive and therapeutic approach to bacterial infections to protect the health of humans and animals. We further assessed the anti-inflammatory effect and mechanism of oral administration of MccJ25 with *in vivo* LPS-induced mouse and cell models of intestinal inflammation.

**Figure 7:**
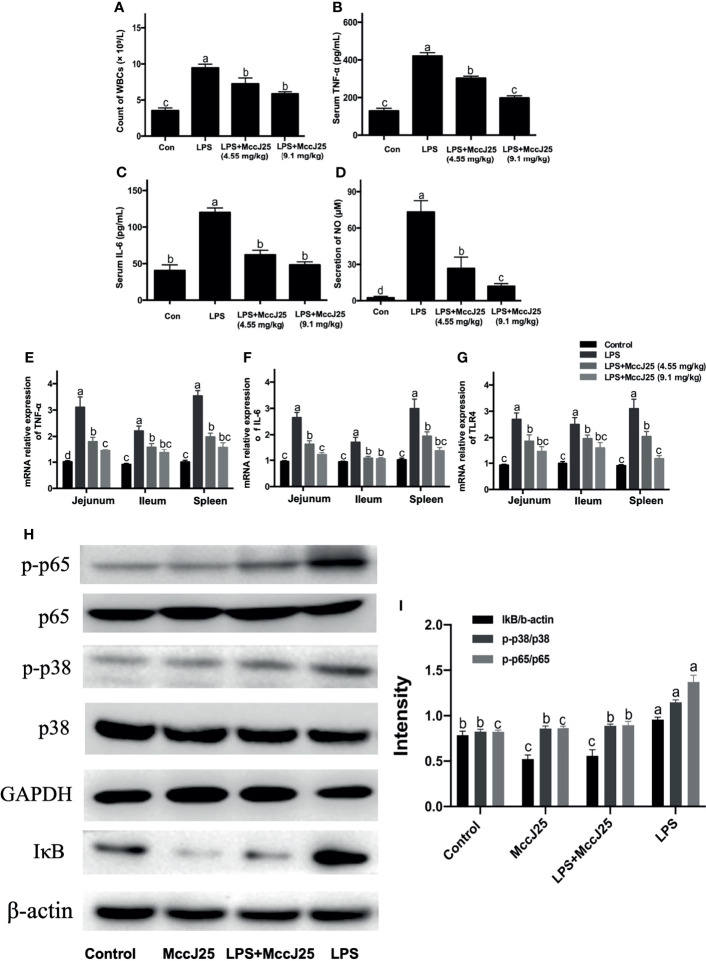
MccJ25 effectively inhibits gut inflammation in LPS-induced mice. **(A-G)** Antimicrobial peptide MccJ25 decreased serum WBC counts **(A)**, TNF-α **(B)**, IL-6 **(C)** secretion, and NO production **(D)**. **(E-G)** AMP MccJ25 inhibited *TNF-α* **(E)**, *IL-6* **(F)**, and *TLR4* **(G)** mRNA expression in the jejunum, ileum and spleen. **(H, I)** MccJ25 possesses an inhibitory function on the NF-κB and MAPK signaling pathways in mice. **(H)** Representative picture of phosphorylated protein levels of NF-κB and p38 MAPK. **(I)** Protein expression of phosphorylated NF-κB and p38 MAPK. Data are expressed as the mean ± standard error of mean of 6 sample replicates. Different superscript lowercase letters within each group indicate significant differences between them (*P* < 0.05).

Then, we evaluated the anti-inflammatory activities of MccJ25. The results showed that the WBC count (8.96 × 10^9^/L) was significantly higher in *E. coli* 0111:B4 LPS-treated BALB/c mice than in untreated mice (**Figure 7A**). In comparison with the results of the *E. coli* 0111:B4 LPS groups, it was found that the application of different concentrations of MccJ25 was able to significantly reduce the counts of leukocytes. Additionally, this finding indicated that the serum levels of TNF-α (**Figure 7B**), IL-6 (**Figure 7C**) and NO (**Figure 7D**) were significantly higher in LPS-challenged mice than in the control group. However, the above indicators reflecting inflammation in MccJ25-treated mice showed a decreasing trend after LPS treatment. Based on the ELISA results, a q-PCR assay was conducted to further evaluate the anti-inflammatory activities of MccJ25. MccJ25 significantly decreased LPS-challenged *TNF-α*, *TLR4* and *IL-6* mRNA expression in the ileum, colon, or spleen (**Figures 7E–G**).

Based on the above experimental findings, we identified changes in the NF-κB and p38 MAPK pathways which are important cellular cascades closely related to inflammation. In comparison with the control treatment group, phosphorylated NF-κB, total IκB and phosphorylated p38 MAPK (**Figures 7H, I**) protein abundance showed a dramatic increase in the LPS group. Critically, MccJ25 alone treated mice group had lower protein expression of phosphorylated NF-κB, total IκB and phosphorylated p38 MAPK than those of treatments (*P* < 0.05). Compared with the LPS-infected mice group, the administration of MccJ25 significantly decreased the abundance of phosphorylated NF-κB, p38 MAPK, and IκB protein expression after LPS challenge (**Figures 7H, I**). Additionally, phosphorylated NF-κB and p38 MAPK protein expression were remarkable decreased in orally administrated with MccJ25 after LPS infection compared with the LPS-treated mice group (*P* < 0.05) (**Figure 7I**)

### MccJ25 Ameliorates LPS-Induced Injury of Organs and Intestinal Tissues

**Figure 8:**
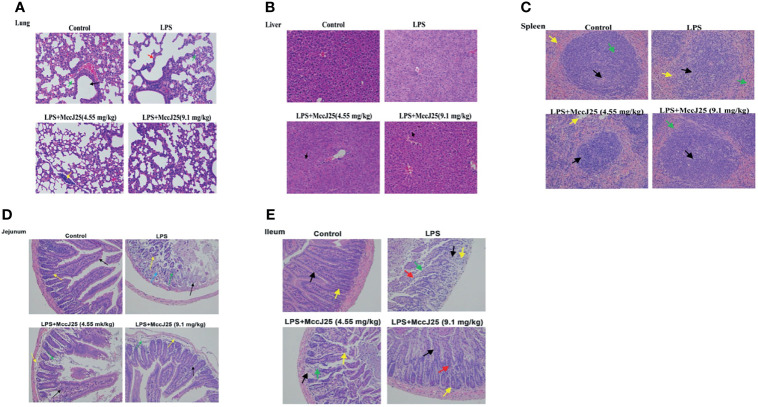
MccJ25 inhibits LPS-induced intestinal and organ injury. **(A-E)** Representative histopathological images of processed lung **(A)**, liver **(B)**, spleen **(C)**, jejunum **(D)**, and ileum **(E)** sections with H&E staining. Images are presented at a magnification of 40 ×. Data are presented as the means ± standard error of mean from 6 biological replicates. Detailed information on different colors of arrows is provided in the **Supplementary Files**.

The lungs of LPS-treated mice were swollen, with significant infiltration of inflammatory cells and shrunken alveoli. MccJ25 appeared to partially reverse swelling and lung damage (**Figure 8A**). Additionally, we observed edema in the liver and spleen of *E. coli* 0111:B4 LPS-treated mice and partial degradation of cytoplasmic proteins but no inflammatory cell infiltration (**Figures 8B, C**). After challenge with *E. coli* 0111:B4 LPS for 30 min, mice that received MccJ25 exhibited a substantially reduced the degree of edema and degradation of cytoplasmic proteins. No inflammatory cell infiltration was detected. These experimental data suggest that MccJ25 belongs to a group of antimicrobial peptides that can exert a very powerful neutralizing effect on the endotoxic activity of BALB/c mice.

Additionally, we found marked villus damage in the intestine (jejunum and ileum) of *E. coli* LPS-treated mice, with sloughing of intestinal epithelial cells (**Figures 8D, E**). Crypts were sparsely arranged, and several crypt epithelial cells were necrotic and sloughed off. The control group, which was characterized by normal villi and intestinal tissue, showed an ordered arrangement of epithelial cells with minimal sloughing, an abundance of distinct crypts and no obvious signs of inflammatory cell infiltration. Administration of MccJ25 (9.1 mg/kg BW), followed by LPS treatment, caused few changes in the villi, crypts and intestinal mucosa compared with the control group. However, slight inflammatory cell infiltration was noted in the lamina propria of the intestinal mucosa. With regard to the lower-dose MccJ25 group (4.55 mg/kg BW), we observed moderate villus damage and epithelial cell sloughing. Moreover, several crypts appeared to be replaced by connective tissue, with a small number of inflammatory cells scattered within the lamina propria.

### Anti-Inflammatory Effects of MccJ25 on RAW 264.7 Cells

**Figure 9:**
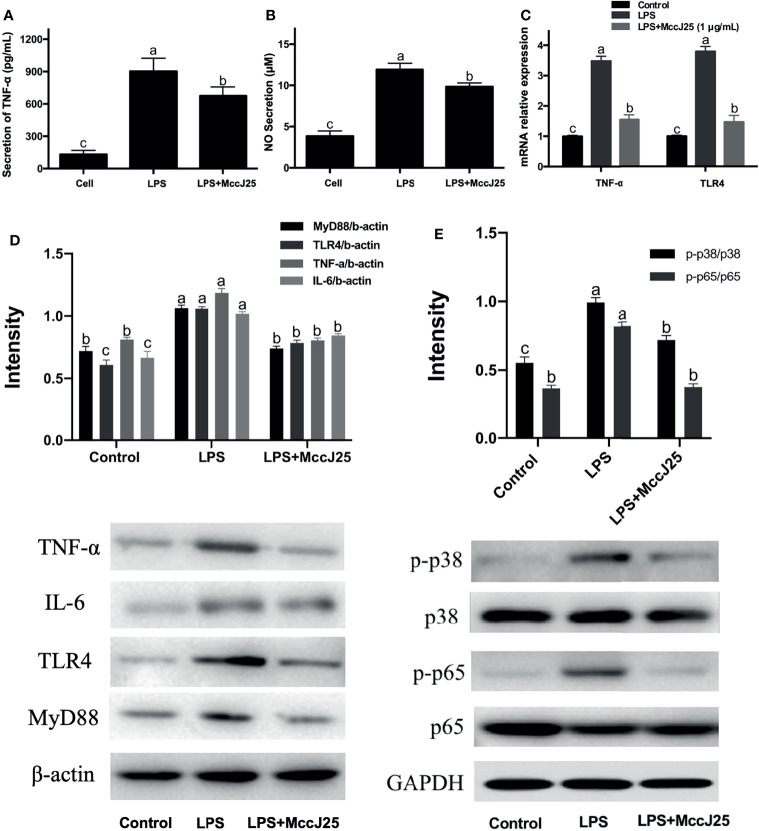
MccJ25 defends against *E coli* LPS O111:B4-induced inflammation in RAW 264.7 cells. **(A, B)** MccJ25 impedes TNF-α **(A)** and NO secretion and production **(B)** in *E coli* LPS-induced RAW 264.7 cells. **(C)** RT–qPCR results indicating intracellular expression of TNF-α and TLR4 in *E coli* LPS-induced RAW 264.7 cells in the presence and absence of MccJ25. Data are presented as the mean ± SEM of 6 biological replicates. MccJ25 significantly reduced the protein expression of TNF-α, IL-6, TLR4, MyD88 **(D)**, phosphorylated NF-κB and p38 MAPK **(E)** in RAW 264.7 cells challenged with LPS by western blotting analysis. Data are shown as the mean ± standard error of mean of 3 biological replicates. Different superscript lowercase letters within each group indicate significant differences (*P* < 0.05).

The ability of MccJ25 to reduce LPS levels in plasmodia and its function to neutralize LPS (1 EU/mL) have driven more scholars to investigate whether MccJ25 can play an inhibitory role in the inflammatory response induced by LPS. To thoroughly clarify the answer to this question, we analyzed the effect of MccJ25 on NO and TNF-α secretion by RAW 264.7 cells induced by *E. coli* LPS. As shown in **Figure 9**, treatment with LPS (1 µg/mL) significantly increased TNF-α production and (**Figure 9A**) and NO (**Figure 9B**) and secretion in RAW 264.7 cells compared to control cells. However, after treatment with MccJ25 (concentration of 1 µg/mL), this increase in secretion was alleviated. In addition, to clarify the nature of the problem, the results of RT-PCR showed that LPS stimulated and directed the expression of *TNF-α* and *TLR-4* in RAW 264.7 cells (**Figure 9C**), while this stimulatory effect was inhibited by MccJ25. Western blotting assay further clarified that LPS-challenged cells had greater MyD88, TLR4, TNF-α and IL-6 protein expression (**Figure 9D**) than control and MccJ25 treated mice group (*P* < 0.05). However, these key upstream regulatory molecules and downstream proinflammatory cytokines were sharply decreased by MccJ25 after LPS-challenged RAW264.7 cells. Surprisingly, there was significant difference between control treatment and MccJ25 treated mice group in the protein expression of regulatory molecules MyD88 and TLR4 (*P* < 0.05).

Important upstream regulatory molecules of the NF-κB signaling pathway are TLR4 and MyD88 (38, 39). Therefore, we further inhibited NF-κB, phosphorylated NF-κB and MyD88 protein expression in RAW264.7 cells. As shown in **Figure 9E**, compared to the control group, phosphorylated NF-κB and MyD88 protein expression in RAW264.7 cells was significantly increased by LPS infection, while MccJ25 treatment significantly decreased p-NF-κB and MyD88 protein expression after LPS challenge for 3 h. The results suggest that MccJ25 could improve LPS-induced macrophage inflammation *in vivo* and *in vitro*. Mechanistically, MccJ25 may downregulate the TLR4-MyD88-NF-κB signaling pathway.

### Pretreated With MccJ25 Effectively Improved Clinical Symptoms Caused by Cocktail of MDR *E. coli* Infection

**Figure 10:**
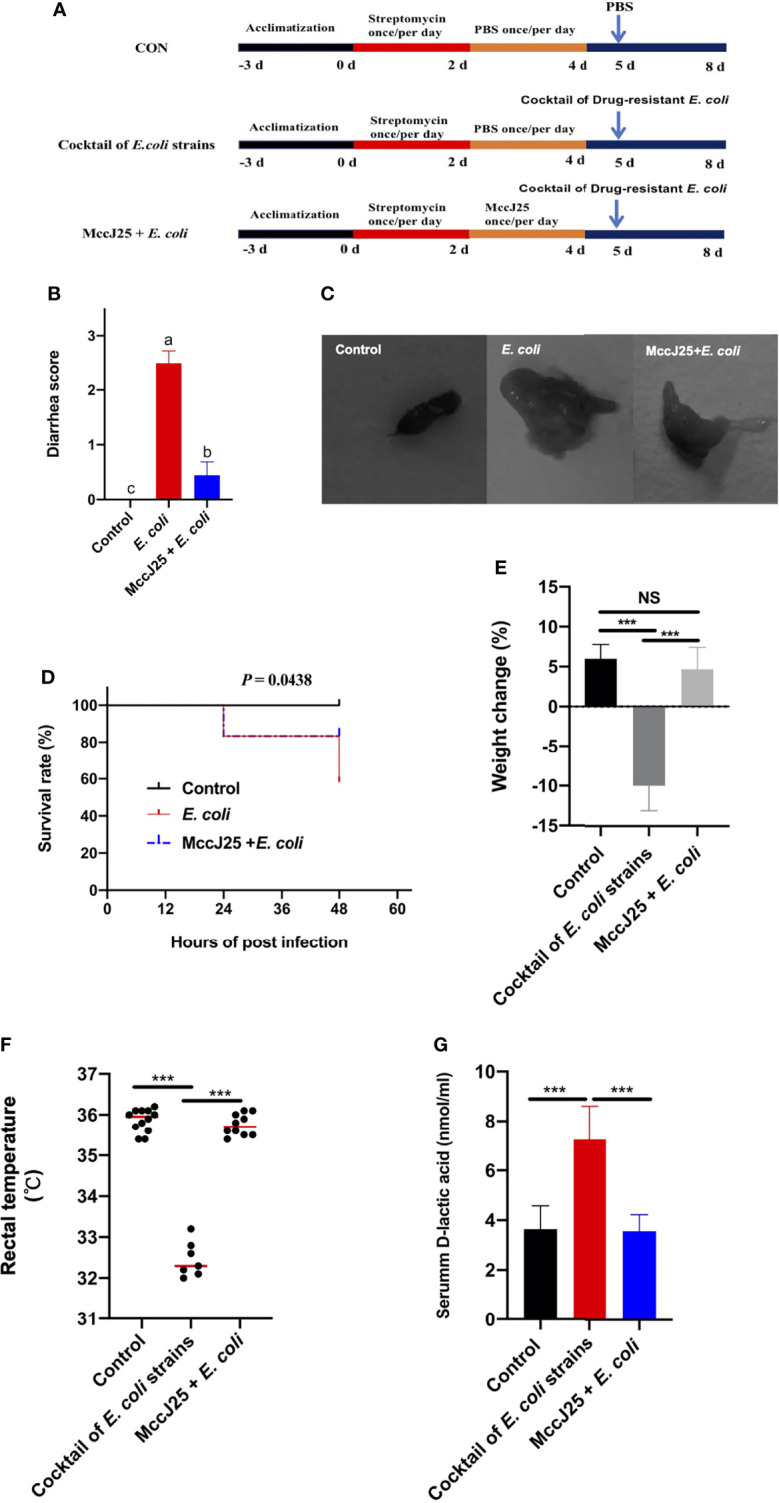
Effects of pretreated with MccJ25 on clinical symptoms and intestinal permeability. Infected mice in the MccJ25 group received 200 μL MccJ25 once/day for 3 d by oral gavage. Mice in the control and infected group were orally administrated with 200 μL sterile PBS. Equal volume PBS containing 10^8^ CFU/mL clinically source cocktail of MDR *E coli* strains was challenged to infect the mice. **(A)** Scheme of the animal trails. **(B)** Evaluation of diarrhea scores (n=12/treatment group). **(C)** Representative image of feces. **(D)** Percentage of survival and body weight changes **(E)** of experimental mice (n = 12/treatment) up to d 3 after cocktail of MDR *E coli* strains infection. **(F)** Record of rectal temperature after cocktail of MDR *E coli* strains infection (n = 12, 7, 10, respectively). **(G)** Serum D-lactic acid concentrations after cocktail of MDR E. coli strains infection (n = 6). Results are indicated as the mean ± standard error of mean. Significant differences were determined using a one-way ANOVA followed by a Tukey’s multiple range test. Statistical difference test of survival curves was analyzed by the Mantel-Cox (log rank). Different superscript lowercase letters within each group indicate significant differences (*P* < 0.05). NS, *P* > 0.5, ***P < 0.001.

Due to the dramatic increase in drug resistance, public health and safety are under great threat. Based on the excellent antibacterial activity of MccJ25 against clinically source MDR bacteria and antiinflammatory responses caused by LPS, we further evaluated the protective ability of MccJ25 against clinically source cocktail of MDR *E. coli* strains in mice. Results showed that cocktail of MDR *E. coli* challenge resulted in a remarkable increase in diarrhea incidence and mortality (**Figures 10B–D**) compared with the uninfected mice group (*P* < 0.05), indicating clinically source MDR *E. coli* strains caused diarrheal disease accompanied with the increase in permeability (**Figure 10G**). The adverse effects were accompanied with the decreased in BW (**Figure 10E**) and rectal temperature (**Figure 10F**). However, mice pretreated with MccJ25 by oral gavage for 3 d efficaciously inhibited cocktail of MDR *E. coli* infection by prolonging the lifespan (*P* < 0.05), increasing BW (*P* < 0.05) and rectal temperature (*P* < 0.05) of mice, and decreasing diarrhea incidence (*P* < 0.05).

### MccJ25 Significant Inhibited Cocktail of MDR *E. coli* Colonization and Translocation

**Figure 11:**
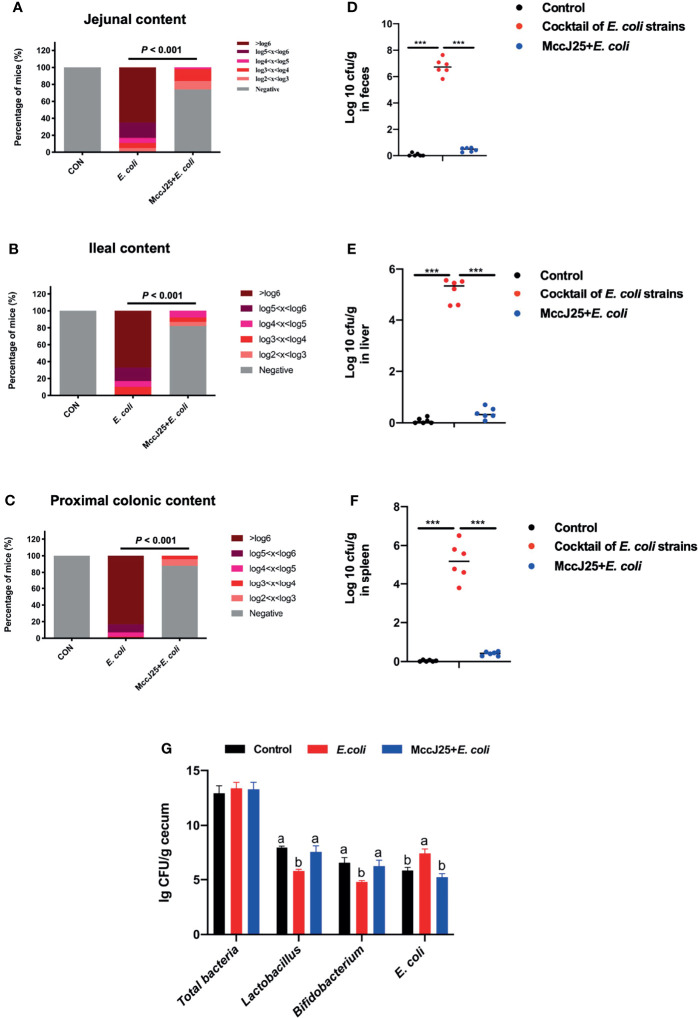
Protective capacity of MccJ25 on the enhancement of host against colonization and invasion of MDR *E coli* strains. Colonization of MDR *E coli* strains in contents of jejunum **(A)**, ileum **(B)**, and proximal colon **(C)**. The nonparametric Mann-Whitney test was used to look for differences in fecal shedding and organ colonization between the infected and MccJ25 groups. The Chi-Square test (likelihood ratio) was performed to examine the frequency of positive animals in various *E coli* levels in intestinal segments. **(D)** Bacterial load in feces. Evaluation of the shedding number of *E coli* in fecal from mice of different treatment group (n = 6) after infection. Assessment of invasion of MDR *E coli* strains in systemic organs including liver **(E)**, spleen **(F)**, and copy numbers of target bacteria in cecum **(G)** were determined at d 3 after infection (n = 6). Assay was performed by counting the bacterial colony using culture-based assay. Results are presented as the mean ± standard error of mean. Significant differences were tested using a one-way ANOVA followed by a Tukey’s multiple range test. ***P < 0.001. Different superscript lowercase letters within each group indicate significant differences (*P* < 0.05).

As indicated in **Figure 11**, clinically source MDR *E. coli* easily colonizes different sites of the jejunum (**Figure 11A**), ileum (**Figure 11B**), and colon (**Figure 11C**) accompanied with dramatic increase in the number of bacteria (*P* < 0.05). Notably, pretreated with MccJ25 significantly reduced the number and colonization of clinically source MDR *E. coli* in the intestines (*P* < 0.05). Furthermore, we found that mice received MccJ25 by oral administration in advance markedly decreased the bacterial load in the feces (**Figure 11D**) compared with the infected mouse group. Subsequently, we clarified the capacity of MccJ25 against the translocation of clinically source MDR *E. coli* colonization in liver and spleen. As shown in **Figures 11E, F**, in the challenged group, the liver (**Figure 11E**), spleen (**Figure 11F**) had greater positive clinically source MDR *E. coli* colonization than the uninfected group. However, compared with the infected group, pretreated with CNM *via* oral gavage significantly reduced the bacteria burden in the spleen and liver, indicating MccJ25 effectively reduced the degree of clinically source MDR *E. coli* colonization and expansion in the gut organs.

Notably, we performed the real-time qPCR to detect the absolute copy numbers of target bacteria including total bacteria, *Lactobacillus*, *Bifidobacterium*, and *E. coli* in cecal contents. Results demonstrated that there is no significant difference in total bacteria copy numbers (*P* > 0.05), but remarkable effects on *Lactobacillus*, *Bifidobacterium*, and *E. coli* were found. Oral administration of MccJ25 significantly increased the copy numbers of *Lactobacillus*, *Bifidobacterium* the sharply decreased the copy number of *E. coli* (*P* < 0.05) as shown in **Figure 11G**.

### MccJ25 Significant Ameliorate Inflammatory Responses in Cocktail of MDR *E. coli* Infected Mouse Model

**Figure 12:**
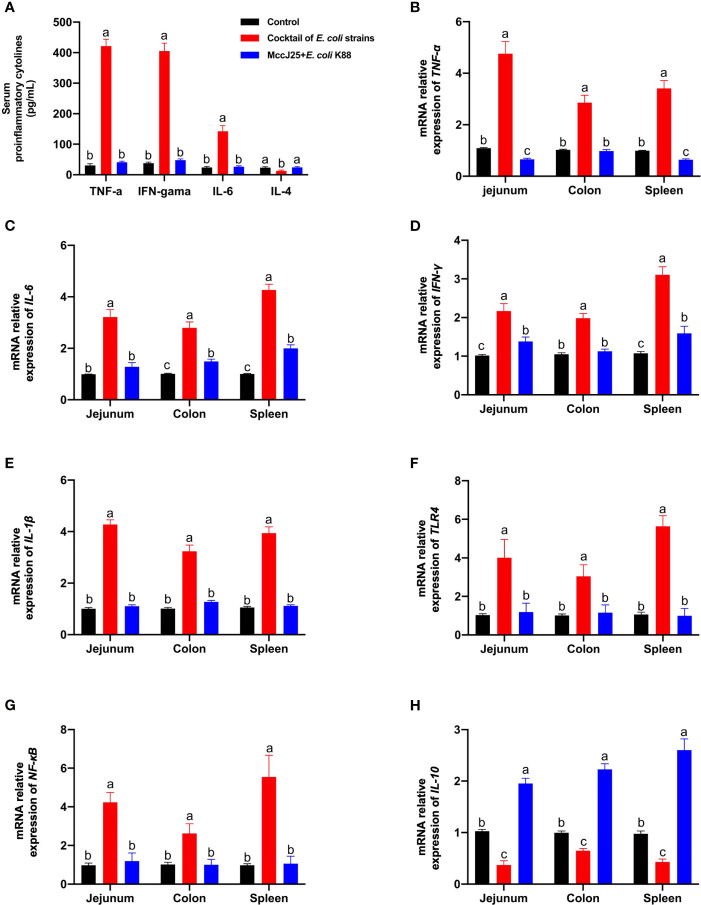
Protective ability of MccJ25 on the inflammatory responses. **(A)** Serum of critical proinflammatory cytokines TNF-α, IFN-γ, IL-6, and IL-4. mRNA expression of proinflammatory markers **(B)** *TNF-α*, **(C)** *IL-6*, **(D)** *IFN-γ*, **(E)** *IL-1β*, **(F)** *IL-10*, **(G)** *TLR4*, and **(H)** *NF-κB*. Infected mice in the MccJ25 group received 200 μL MccJ25 once/day for 3 d by oral gavage. Mice in the control and infected group were orally administrated with 200 μL sterile PBS. Equal volume PBS containing 10^8^ CFU/mL clinically source cocktail of MDR *E coli* strains was challenged to infect the mice. Data are given as the mean ± standard error of mean (n = 6). Differences were analyzed using a one-way ANOVA followed by a Tukey’s multiple range test. Different superscript lowercase letters within each group indicate significant differences (*P* < 0.05).

As shown in **Figure 12A**, clinically source MDR *E. coli* strains infection caused serious systemic inflammatory response by sharply increasing the serum proinflammatory cytokines, such as TNF-α, IFN-γ, IL-6 (*P* < 0.05), and a significant reduction in IL-4 levels (*P* < 0.05) compared with the control group. As we expected, mice received MccJ25 by oral administration in advance had lower proinflammatory cytokines (*P* < 0.05) and higher IL-4 (*P* < 0.05) respectively than infected mice group. Moreover, no significant differences in inflammatory factors were observed between uninfected group and MccJ25 treated mice group. (*P* > 0.05).

Consistent with the ELISA results, qPCR results also showed that Th1/Th2 cytokines *TNF-α* (**Figure 12B**)*, IL-6* (**Figure 12C**)*, IFN-γ* (**Figure 12D**), *IL-1β* (**Figure 12E**), as well as important regulation moleculars *TLR4* (**Figure 12F**), and *NF-κB* (**Figure 12G**) mRNA expression in the jejunum, colon and spleen in clinically source MDR *E. coli* strains infected mice were significantly increased, or Th2 cytokine *IL-10* (**Figure 12H**) was remarkable decreased compared with the uninfected mice (*P* < 0.05). However, mice were orally administrated with MccJ25 had lower proinflammatory indicators (*P* < 0.05) and high anti-inflammatory cytokine *IL-10* (*P* < 0.05) than those of infected mice. Additionally, pretreated with MccJ25 to mice had similar to the low *TNF-α* (**Figure 12B**) and *IFN-γ* (**Figure 12D**) in the colon, and *IL-6* (**Figure 12C**) in the jejunum, and *IL-1β* (**Figure 12E**), *TLR4* (**Figure 12F**), and *NF-κB* (**Figure 12G**) mRNA expression in the jejunum, colon and spleen. However, compared with uninfected mice group, significant differences in the mRNA expression of *IL-10* in jejunum, colon, and spleen (**Figure 12H**) and *TNF-α* in jejunum and spleen (**Figure 12B**) were observed in treated MccJ25 mice group after clinically source MDR *E. coli* strains infection. These findings evidenced that MccJ25 harbors a significant improved defense response against the Inflammatory bowel disease caused by pathogenic microorganisms, especially multi-drug resistant bacteria. These data evidenced that MccJ25 could inhibit enteric pathogens, reduce enterobacterial blooms, and decrease inflammation in the inflamed gut.

## Discussion

Our findings in the current study demonstrate that MccJ25 has strong antibacterial/bactericidal activity against the sensitive bacterium ETEC by disrupting the cell membrane. As an excellent antimicrobial agent, MccJ25 is stable under physiological conditions. Furthermore, risk assessment of MccJ25 revealed by cytotoxicity and resistance indicates that MccJ25 is unlikely to cause side effects, and the idea that MccJ25 can be useful in clinical practice is recognized. Subsequently, our study further demonstrates the MccJ25 harbors a significant improved defense response against the Inflammatory bowel disease caused by pathogenic microorganisms, especially multi-drug resistant bacteria infection, and LPS. These findings in the present study evidenced that promising and potential application of recombinant MccJ25 as an antimicrobial/antiendotoxin peptide to treat inflammatory bowel disease caused by LPS or pathogens.

Most commensal pathogens are gram-negative bacteria of the Enterobacteriaceae family. Some of these pathogens, especially clinically source MDR bacteria, are the leading causes of morbidity and mortality in young animals and humans (40, 41). For example, pathogens of the Enterobacteriaceae family, such as *E. coli* or *Salmonella*, have caused substantial losses to livestock, agriculture and poultry industries with significant negative impacts on the food production chain and various diseases in humans. Because humans and animals have been selectively using the effects of antibiotics, this has driven the birth of antibiotic-resistant microorganisms (42–44). In the post antibiotics era, it is critical and urgent to develop novel antimicrobial alternative to traditional antibiotics to target the bacterial infection. One class of molecules that has not gained prevalence but holds particular promise as clinical antibiotics is ribosomally encoded AMPs.

It has been suggested that natural, natural MccJ25 possesses very potent antibacterial activity with MICs in the nanomolar range (between 2 and 50 nM) against some of the more common gram-negative bacteria, such as *Salmonella*, *E. coli*, and *Shigella* (20, 22, 45). Ribosomally synthesized microcins have strong bactericidal activity within the pico- or nanomolar concentration range and play key roles in innate immunity (46–48). As potential antimicrobial agents, few microcins have been commercially applied. Various assays must be performed to establish the safety of MccJ25 (cytotoxicity, hemolysis, and resistance) before its use in the food, medicine and veterinary industries. In our previous studies, we engineered and constructed a highly efficient expression system for the large-scale production of MccJ25 and proved that the molecular weight and amino acid sequence of MccJ25 were consistent with those of natural MccJ25 (24) Although MccJ25 exerts a narrow spectrum of antimicrobial activity against *E. coli and Salmonella*, MccJ25 harbors different antibacterial activities toward different species and serotypes of bacteria (26). We need further evaluated the activity of MccJ25. In the present study, the results of antimicrobial assays demonstrated that MccJ25, similar to natural MccJ25, possessed remarkable antimicrobial/bactericidal activity against standard gram-negative bacterial and clinically relevant veterinary drug-resistant *E. coli* and *Salmonella*. Anti-adherence assay (**Figure S1**) and Live/dead (**Figure 2**) results showed that MccJ25 exerted strong bactericidal activity and inhibited pathogen adherence. Once penetrating bacterial cells, MccJ25 causes severe loss of inner membrane integrity, resulting in bacterial death. Sytox 9 assays further demonstrated that MccJ25 can disrupt ETEC K88 membrane integrity by binding DNA. The ability of MccJ25 to block DNA migration on gels at a ratio of 2:1 confirms its ability to affect the normal synthesis of bacterial DNA. We hypothesized that once penetrated through the bacterial cell, MccJ25 would be able to achieve the ultimate goal of killing bacteria by inhibiting RNA polymerase activity (35, 49). Some studies have also been described; natural MccJ25 was found to permeabilize the cytoplasmic membrane, thereby disrupting the electrochemical gradient in *Salmonella* Newport. Natural MccJ25 can also induce superoxide formation and disrupt the membrane respiratory chain in *E. coli*, suggesting that MccJ25 may have more than one intracellular target and mediate several independent mechanisms in different sensitive strains (16, 35). The present study was in line with our previous report that MccJ25 can kill *S. pullorum* by changing the membrane morphology and disrupting the cytoplasmic membrane (26). MccJ25 has the potential to be used as an antimicrobial agent and as a bioactive compound in nutritional, animal or agricultural applications.

The results of the data obtained herein demonstrate the feasibility of MccJ25 in controlling pathogens. However, the inhibition of antimicrobial peptide activity by protease digestion hinders further development of safe and effective peptides for clinical application. The results of numerous studies have shown that a key constraint to the development of antimicrobial peptides is their low stability at different temperatures and pH values and in gastric and pancreatic enzymes. This feature largely hinders the use of most peptides in real life as drugs or feed additives for animals and humans. Moreover, AMPs are also very unstable and easily degraded (26, 27, 35). In this paper, the bactericidal activity of the biogenic source MccJ25 toward *E. coli* K88 cells was not affected when the temperature and pH were changed or after treatment with pepsin, trypsin or chymotrypsin. These findings are generally consistent with those of previous authors, indicating that MccJ25 maintains a steady state under complex conditions (50, 51). In addition, the *in vitro* activity of MccJ25 was evaluated in a simulated gastrointestinal environment and in serum. This experiment was performed to determine the fate of MccJ25 after oral administration. Our results show that MccJ25 can exert antimicrobial activity in mouse serum, stomach and intestine. This observation is consistent with previous reports showing that several AMPs and micro/nanobiomaterials have stable bactericidal effects that are retained in SGF, SIF, and serum (27, 52). The results of this study are also consistent with those of our previous study, which indicated that recombinant MccJ25 was fairly stable to kill foodborne pathogens (26).

Microcins, AMPs, are a large class of antimicrobial agents that can be employed in the treatment of various infectious diseases. Although natural AMPs have been shown by several studies to possess a good ability to overcome multidrug resistance, some of the problems, including a lack of key points on systemic toxicity risk, may be important factors hindering the evolution of peptides to antimicrobial drugs. Cytotoxicity, including the hemolysis of erythrocytes, is one of the main factors limiting the practical application of AMPs. Therefore, it is crucial to have a clear understanding of the cytotoxic effects of peptides (52–56). In this study, MccJ25 was hypothesized to fight against infectious diseases with weak hemolytic effects or little cytotoxicity. In our previous study, we found that MccJ25 was not cytotoxic toward IPEC-J2 cells (29). For the application of MccJ25 as a drug or an antimicrobial agent in food, humans, and veterinary use, it is critical to further investigate the cytotoxicity on different cell lines. Although we have shown that MccJ25 did not cause cytotoxicity to intestinal porcine epithelial cells and induced toxicity risk toward mice at low levels (24), to avoid specificity of MccJ25 toward the different mammalian cells and observe comparative cytotoxicity assessment of MccJ25, the type of cells needs to be considered. Consistent with our previous study (29), the results of the current study demonstrated that MccJ25 remained nontoxic to human colon cancer Caco-2 cells and mouse macrophage RAW264.7 cells even when the concentration of MccJ25 was very high (512 μg/mL). The results of hemolytic assays showed that MccJ25 did not induce hemolysis in mRBCs and pRBCs, reflecting the potential of MccJ25 as an alternative in various industrial domains. However, the reason is not clear yet. We hypothesize that the very low cytotoxicity of MccJ25 is due to its key lasso structure properties. An appropriate balance among these structural parameters is critical to the antimicrobial activity of AMPs (27). Therefore, in future studies, we need to investigate the mode of action of low cytotoxicity activity of MccJ25. Additionally, MccJ25 may also be selective for the anionic component of microbial cell membranes but not for the zwitterionic component of mammalian cell membranes.

The mutation rates induced by AMPs are low, suggesting that they can potentially evade multidrug resistance because their mechanism of action involves the destruction of microbial membranes (9–12). For example, in a study by Navon-Venezia et al. (57), it was shown that repeated exposure of gram-positive bacteria to different AMPs did not show significant changes in the MIC of 10-15 bacterial channels at subminimal inhibitory concentrations. However, ampicillin, ciprofloxacin, and kanamycin can significantly increase mutation rates (58). The mutation rate is a key indicator to determine the rate of microbial adaptation to antibiotic stress (59). Compared to the negative control, MccJ25 did not increase the mutation rate in bacteria, suggesting that MccJ25 may behave similarly to other AMPs that cause pathogen death. It has also been indicated that sublethal concentrations of antibiotics have the potential to induce multidrug resistance, attracting the interest of many scholars in the prophylactic use of antibiotics in food production animals for the purpose of growth promotion (37, 58). As shown in the results, there was no increase in MIC when pathogens were killed using MccJ25 (0.5 × MIC), suggesting that MccJ25 at sublethal concentrations may not cause multidrug resistance. Therefore, our results support the prophylactic use of MccJ25 in different domains including food, clinical, and animals.

The immune effect on bacterial infection refers mainly to the production of cytokines and chemokines through various immune cells (e.g., macrophages, monocytes, and NK cells) (60–63). Bacterial infections or LPS infection are able to cause damage to the intestinal microecology and barrier function. When the intestinal epithelial barrier faces damage, the intestinal microbiota also assumes a more severe risk of inflammation and infection. At this stage, morbidity and mortality caused by infections with enterotoxigenic bacteria such as *E. coli*, *Staphylococcus aureus*, and *Pseudomonas aeruginosa* are mainly related to the stimulated inflammatory response (64–69). Previous study results showed that the administration of MccJ25 was able to significantly reduce the number of *E. coli* in mice treated with *E. coli* K88 cells (30, 32). Shang et al. (70) showed that MccJ25 effectively ameliorated the intestinal inflammatory disease caused by DSS induction *via* improving gut microbiota and modulating Th1/Th2 immune function. Thus, MccJ25 can be applied as an anti-inflammatory agent. We further investigated anti-LPS challenge-induced intestinal inflammation.

Another unfavorable feature of biomaterials comes from the immunogenicity formed by the interaction between AMPs and the host organism through the Toll-like receptor (TLR) pathway, which is mainly seen in hypercytokinemia or cytokine storms and causes severe inflammation (39, 70). LPS on gram-negative bacterial membranes induces inflammation, which is a physiological manifestation of various diseases, such as septic shock induced by LPS- or lipoteichoic acid (LTA) (71–73). These responses significantly indicate an enhanced effect of the expression of inflammatory mediators such as TLR4 and nitric oxide (NO)-related proteins, such as inducible NO synthase. Based on the interaction with TLR4 on macrophages, LPS stimulates the production of NO and proinflammatory cytokines, which contain TNF-α and interleukin-1β (IL-1β) (74, 75). In the current study, we used the limulus amebocyte lysate method (0.5-128 μg/mL MccJ25 and 1 EU/mL LPS) and observed that MccJ25 neutralized LPS in a dose-dependent manner, with approximately 50% and 93% neutralization at 1 and 128 μg/mL, respectively. Based on the results of the antimicrobial activity assay, MccJ25 exhibited good antimicrobial activity, minimal cytotoxicity, and the best affinity for binding to LPS. Observations showed that serum levels of inflammatory markers (such as NO, TNF-α and IL-6) and LPS were significantly reduced when *E. coli* LPS-stimulated mice were treated with MccJ25 after challenge with LPS for 30 min. MccJ25 made by biosynthetic methods elevated the probability of survival in mice while relieving stress on the lungs and liver. The results also showed that mice were challenged with LPS for 30 min. treatment of LPS-challenged mice with MccJ25 significantly reduced the mRNA expression of TNF-α, TLR4, and IL-6 in the jejunum, ileum and spleen and alleviated inflammation and disease damage at these sites. In addition, observations showed that the production of NO, TNF-α and IL-6 stimulated by *E. coli* LPS in RAW 264.7 cells for 3 h was inhibited by MccJ25. The results of RT–PCR and Western blotting also showed that MccJ25 could inhibit the expression levels of TNF-α, IL-6 and TLR4. In future studies, the mode of action of MccJ25 neutralizing LPS, resulting in LPS-antagonizing effects, needs to be studied.

TLR4 and MyD88 are important regulatory molecules upstream of the NF-κB signaling pathway. NF-κB signaling regulates cytokines and cells involved in the inflammatory process (71–73), and LPS activates NF-κB-IκB and MAPK *via* interactions with the TLR4-MyD88 signaling pathway, driving a large and dramatic expression of downstream proinflammatory factors and leading to an inflammatory response. Therefore, NF-κB-IκB and MAPK are considered to be important active factors regulating inflammatory gene expression. In the present study, we found that MccJ25 significantly reduced key proinflmmatory markers, such as TNF-α, IL-6, NF-κB, TLR4 and MyD88 protein expression. Mechanistically, MccJ25 suppressed proinflammatory responses *in vitro* and *in vivo* by decreasing the production of proinflammatory markers *via* down-regualtin the activation of the NF-κB-IκB and MAPK signaling pathways. The regulation of the NF-κB-IκB and MAPK signaling pathways may be due to the antimicrobial mechanism of MccJ25 and LPS neutralization because the TLR4 is a key LPS receptor. Consistent with the results of this experiment, the antimicrobial peptides LFP-20 and CWA directly inhibited the MyD88/NF-κB signaling pathway and alleviated LPS-induced macrophage inflammation (76–78). Therefore, in addition to the antibacterial activity and LPS neutralization, the above results suggest that MccJ25 can also downregulate the TLR4-MyD88-NF-κB-IκB and MAPK signaling pathways to alleviate intestinal inflammation. Additionally, in future studies, it will be important to investigate the mechanism by which MccJ25 binds and neutralizes LPS and other potential anti-inflammatory mechanisms, such as enhancing macrophage phagocytosis and chemotaxis.

Microcin J25 has attracted considerable interest because of the high stability of its lasso structure, which can be used as potential antimicrobial agent, but it has not been commercially applied (8, 16, 17). In our previous study, we showed that MccJ25 can be produced on a large-scale and harbors narrow-spectrum antibacterial activity targeting Gram-negative bacteria. Low concentration of MccJ25 did not cause *in vivo* toxicity risk and modulated intestinal health including improving gut microbiota, enhancing barrier integrity, and modulating immune function in different models (bacterial infection and DSS infection) and administration routes; however, different assays must be performed to evaluate the safety and toxicity of MccJ25 (cytotoxicity and hemolysis) before its application in the food, medicine and veterinary industries (24, 26, 29, 31, 79–81). Moreover, the potential development of bacterial resistance to MccJ25 upon repeated administration requires evaluation. Convincing evidence is needed to prove the pleiotropic functions of biosynthetic MccJ25 not only in the eradication of pathogens from the gastrointestinal tract but also in the maintenance of homeostasis and in the alleviation of inflammatory responses *in vitro* and *in vivo*.

Until now, there is no doubt that highly serious issues in public health is antibiotic resistance. We have officially entered the post-antibiotics era. Clinically source MDR bacterial infections are the serious killer toward human and animal health (82, 83). For instance, it is estimated that antibiotic resistance will cause deaths more than 10 million/year, the number of deaths will be surpassing cancers by 2050 (84, 85). The serious issue of clinically source MDR bacterial infection needs to be addressed, new antimicrobial stewardships need to be developed urgently to solve the problem of clinically source MDR bacterial infections. In the current study, we found that MccJ25 exerted strong antimicrobial activity against clinically source MDR *E. coli* and *Salmonella* strains (**Table 2**). Based on the results and our previous studies, we tested the ability of anti-infection using cocktail clinically source MDR *E. coli* strains in mice model. As we expected, we uncovered that pretreated with MccJ25 effectively protected mice to defense against cocktail clinically source MDR *E. coli* strains infection by prolonging the lifespan, increasing body weight, and decreasing diarrhea incidence. These results are line with previous studies that AMPs significantly improved clinical symptoms by regulating immune function and improving gut microbiota composition, thereby inhibiting colonization and translocation of pathogens using infective animal model (32, 38, 39, 77). Consistent with these previous reports, in this study, we found that MccJ25 harbors a significant improved defense response against the Inflammatory bowel disease caused by pathogenic microorganisms, especially multi-drug resistant bacteria. These data evidenced that MccJ25 could inhibit enteric pathogens, reduce enterobacterial blooms, and decrease inflammation in the inflamed gut.

For these reasons, we conclude that administration of MccJ25 shows strong bactericidal activity and can protect hosts defense against clinically source MDR *E. coli* strains infection *in vivo*. Additionally, MccJ25 may alleviate this pathogens or LPS-induced inflammation caused by gram-negative bacteria through endotoxin neutralization ability and TLR4-MyD88-NF-κB and p38 MAPK signaling pathways to regulate the Th1/Th2 immune balance.

## Conclusions

In the current study, we demonstrated that MccJ25 exerted significant antimicrobial activity against gram-negative bacteria and clinically relevant veterinary drug-resistant *E. coli* and *Salmonella* with different MICs and MBCs, indicating that MccJ25 exerts different bactericidal activities targeting different pathogens *via* permeabilizing the bacterial membrane. When administered orally, MccJ25 will be under the influence of barriers that may cause the side effects on stability and biological activity. Our findings indicated that MccJ25 showed excellent stability in real-word conditions. Comprehensive safety and toxicity risk assessment uncovered that MccJ25 did not cause cytotoxicity, hemolysis, or showed low propensity to induce resistance. Under the basis of strong bactericidal activity and safety, in LPS-challenged *in vitro* and *in vivo* models, MccJ25 showed anti-inflammatory activity. Additionally, therapeutic potential data showed that MccJ25 significantly inhibited cocktail of MDR *E. coli* strains infection caused the gut inflammation by decreasing the critical proinflammatory cytokines *via* TLR4-MyD88-NF-κB or p38 MAPK pathways, and bacteria burden *via* reducing colonization, translocation of bacteria. These set of phenomena observed in the experiments of this study provides a solid basis for advancing the development of AMP MccJ25 as promising active anti-infective agents and anti-inflammatory agents in the battle against drug-resistant infections caused by pathogens or LPS.

## Data Availability Statement

The original contributions presented in the study are included in the article/**Supplementary Material**. Further inquiries can be directed to the corresponding author.

## Ethics Statement

This study including two infected mice experiments was conducted mainly following the provisions in the Chinese Code of Welfare and Ethics for Laboratory Animals. This experimental protocol was approved by the Institutional Animal Care and Use Committee of China Agricultural University (CAU No. AW04101202-1-1) and confirmed by the Regulations for the Administration of Affairs Concerning Experimental Animals of the State Council of the People’s Republic of China (No. SYXK(Jing) 2015-0028).

## Author Contributions

HY wrote the manuscript. HY, XZ, and SQ clarified the overall idea of this study. HY and LS were mainly responsible for animal research. HY and GY were mainly responsible for the cells study. HY and ZD analyzed the data. HY and XZ contributed to the revision of the manuscript. XZ and SQ guided the experiments. All authors contributed to the writing of the thesis and finally summarized and integrated it into the final draft.

## Funding

This study was conducted with the support and assistance of the National Natural Science Foundation of China (Major Project; Grant No. 32030105), Chongqing Rongchang Agricultural and Animal Husbandry High-tech Industry Research and Development Project (cstc2019ngzx0019) and Beijing Livestock Innovation Team of Modern Agriculture Industry Technological System.

## Conflict of Interest

The authors declare that the research was conducted in the absence of any commercial or financial relationships that could be construed as a potential conflict of interest.

The reviewer XW declared a shared affiliation, with no collaboration, with the authors to the handling editor at the time of the review.

## Publisher’s Note

All claims expressed in this article are solely those of the authors and do not necessarily represent those of their affiliated organizations, or those of the publisher, the editors and the reviewers. Any product that may be evaluated in this article, or claim that may be made by its manufacturer, is not guaranteed or endorsed by the publisher.

## Abbreviations

MccJ25, microcin J25; AMPs, antimicrobial peptides; MIC, minimum inhibitory concentration; MBC, minimum bactericidal concentration; *E. coli*, *Escherichia coli*; *S.* typhimurium, *Salmonella* typhimurium; *S.* pullorum, *Salmonella* pullorum; ETEC, enterotoxigenic *E. coli*; LDH, lactate dehydrogenase; IPEC-1, porcine epithelial cell line 1; GAPDH, glyceraldehyde-3- phosphate dehydrogenase; SGF, simulated gastric fluid; SIF, simulated intestinal fluid; BSA, bismuth sulfite agar; PBS, phosphate buffered saline; CFU, colony forming units; LPS, lipopolysaccharide; IL, interleukin; IFN, interferon; TNF-α, tumor necrosis factor-α; TLR4, Toll-like receptor 4; MyD88, myeloid differentiation factor 88; IkB, nuclear factor kappa-B kinase; NF-kB, nuclear factor kappa-B; MAPK, mitogen-activated protein kinase; MDR, Multi-drug resistant.
