# Supplementary material for: Biosynthetic Microcin J25 Exerts Strong Antibacterial, Anti-Inflammatory Activities, Low Cytotoxicity Without Increasing Drug-Resistance to Bacteria Target
Source: Front Immunol. 2022 Feb 18;13:811378. doi: 10.3389/fimmu.2022.811378 (PMC8894198; doi:10.3389/fimmu.2022.811378)
Supplement: Supplementary file 1 [file DataSheet_1.docx]

**Supplementary Materials**

**Microcin J25 Exerts Strong Antibacterial, Anti-Inflammatory Activities, Low Cytotoxicity without Increasing Drug-Resistance to Bacteria Target**

*Haitao Yu^1,2^, Lijun Shang^1,3^, Guangxin Yang^1,3^, Ziqi Dai^1,3^, Xiangfang Zeng^1,3^* and *Shiyan Qiao^1,3,*^*

^1^*State Key Laboratory of Animal Nutrition, Ministry of Agriculture and Rural Affairs Feed Industry Center, China Agricultural University, Beijing 100193, P.R. China;*

*^2^Institute of Systems Biomedicine, Department of Immunology, School of Basic Medical Sciences, Beijing Key Laboratory of Tumor Systems Biology, Peking University Health Science Center, Beijing, China;*

^3^*Beijing Key Laboratory of Biofeed additives, China Agricultural University, Beijing 100193, P.R. China*

**Corresponding authors: Shiyan Qiao, Professor, State Key Laboratory of Animal Nutrition, College of Animal Science and Technology, China Agricultural University*

*E-mail: qiaoshiyan@cau.edu.cn*

*Tel: +86 10-62733588*

*Fax: +86 10-62733688*

**Results**

**SUPPLEMENTARY MATERIALS AND METHODS**

**IPEC-1 cells culture**

Intestinal epithelial cell and murine cell line macrophages, RAW264.7 cells were seeded into Dulbecco’s modified Eagle’s medium (DMEM) F12 supplemented with 10% fetal bovine serum (FBS) on a 96-well plate at a density of 2 × 10^4^ cells/mL until 89-90% confluence.

**Protective ability**

Capacity of against *E. coli* K88 adhesion and colonization of IPEC-1 cells was evaluated. IPEC-1 cells were cultured with or without MccJ25 for 3 h in the absence or presence of *E. coli* K88 for 2 h. The *E. coli* K88 adhesion and colonization in the IPEC-1 cells was determined. *E. coli* K88 (mean CFU/ml ± SEMs) were determined by plate counts, and determined from three individual experiments.

**Killing curves and Live/dead assay**

A purified single colony of *E. coli* K88 was inoculated into 5 mL of LB. Then, culture was incubated at 37◦C with shaking at 200 rpm overnight. The next day, the culture was diluted 1:100 in fresh LB and incubated at 37◦C with shaking at 200 rpm again. To test antimicrobial activity, the bacteria were grown until they reached log phase. Approximately 5 × 10^5^ or 10^8^CFU/mL *E. coli* K88 were inoculated into 2 mL of LB containing different levels of the MccJ25 (0, 1 × MIC, 2 × MIC, 4 × MIC or 4 × MIC). The cultures were serially diluted with sterilized saline water and plated on LB agar at different hours. The plates were incubated at 37◦C overnight to count colony. Colonies were counted to determine CFU/mL. The experiment was conducted with two replicates in three independent trials. A Live/Dead BacLight Bacterial Viability Kit 7 (Molecular Probes, Inc., Eugene) was used to test bacterial viability. Briefly, 5 × 10^5^CFU/mL *E. coli* K88 were inoculated into 1 mL of LB in the presence of MccJ25 at the MIC level. *E. coli* K88 cultures were incubated at 37◦C for 2 h and then incubated in the dark at ambient temperature with SYTO 9 and propidium iodide for 15 min. Bacteria were observed using a fluorescence microscope (EVOS XL Cell Imaging System).

**SUPPLEMENTARY TABLES**

**Table S1. Quantitative Real-Time polymerase chain reaction (qRT-PCR) primers sequences applied in this study**

| Genes |  | Sequence (5'-3') | Size (bp) |
| --- | --- | --- | --- |
| *GAPDH* | Forward | GAGAAACCTGCCAAGTATGATGAC | 212 |
|  | Reverse | TAGCCGTATTCATTGTCATACCAG |  |
| *TNF-α* | Forward | CCACGCTCTTCTGTCTACTG | 169 |
|  | Reverse | ACTTGGTGGTTTGCTACGAC |  |
| *IL-6* | Forward | GAGTCACAGAAGGAGTGGCTAAGGA | 106 |
|  | Reverse | CGCACTAGGTTTGCCGAGTAGATCT |  |
| *IL-10* | Forward | GCCTTATCGGAAATGATCCA | 158 |
|  | Reverse | TTTTCACAGGGGAGAAATCG |  |
| *IL-1β* | Forward | GCCTTGGGCCTCAAAGGAAAGAATC | 183 |
|  | Reverse | GGAAGACACAGATTCCATGGTGAAG |  |
| *IFN-γ* | Forward | CCATCGGCTGACCTAGAGAA | 162 |
|  | Reverse | AACAGCCAGAAACAGCCATG |  |
| *TLR4* | Forward | GCTTTCACCTCTGCCTTCAC | 169 |
|  | Reverse | TGCCATGTTTGAGCAATCTC |  |
| *NF-κB* | Forward | CCAGAAGAGGGTGTCAGAGC | 226 |
|  | Reverse | TCGAAATCCCCTCTGTTTTG |  |
| Total bacteria | Forward | ACTCCTACGGGAGGCAGCAG |  |
|  | Reverse | ATTACCGCGGCTGCTGG |  |
| *Bifidobacterium spp* | Forward | CGCGTCCGGTGTGAAAG |  |
|  | Reverse | CTTCCCGATATCTACACATTCCA |  |
| *Lactobacillus spp* | Forward | GAGGCAGCAGTAGGGAATCTTC |  |
|  | Reverse | GGCCAGTTACTACCTCTATCCTTCTTC |  |
| *E. coli* | Forward | CATGCCGCGTGTATGAAGAA |  |
|  | Reverse | CGGGTAACGTCAATGAGCAAA |  |


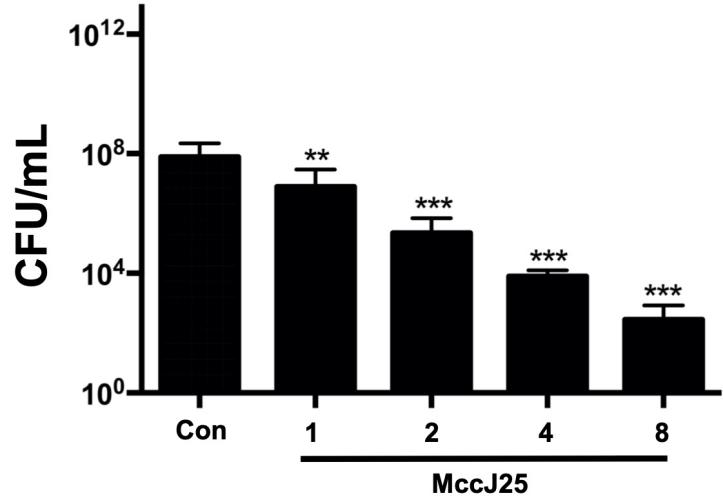


Figure S1. MccJ25 enhances intestinal epithelial cell to against *E. coli* K88 infection. 1, 2, 4, 8 × MIC of MccJ25 significantly protects IPEC-1 against *E. coli* K88 adhesion and colonization, respectively. IPEC-1 cells were cultured in the presence or absence of 1, 2, 4, 8 × MIC of MccJ25 for 3 h, then cells were treated with *E. coli* K88 for 2 h. The numbers of *E. coli* K88 were counted. *E. coli* K88 (mean CFU/ml ± SEMs) were determined by plate counts from from 6 biological replicates. ***P* ≤ 0.01, ****P* ≤ 0.001.


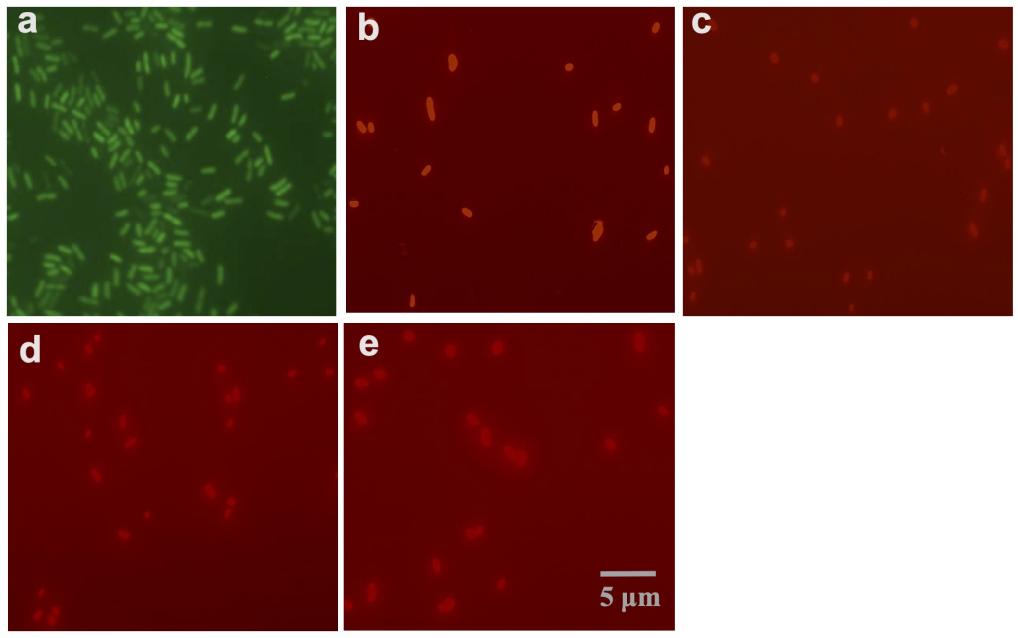


**Figure S2.** Bactericidal activity assay of MccJ25 by Live/dead assay. (a). control; (b-d). 1, 2, 4, 8 × MIC of MccJ25, respectively.

**Figure 8 Legends:**

(A) The black arrow shows the bronchial structure; the green arrow shows the alveolar structure; the red arrow shows the proliferative alveolar wall; the yellow arrow shows the focal infiltration of inflammatory cells (lymphocytes and monocytes). (B) The black arrow shows a small amount of cell edema and light staining of cytoplasmic protein. (C) The black arrow shows the infiltration of nuclear fragments and spleen inflammatory cells; the yellow arrow shows the unclear boundary of red and white pulp, the increase of red pulp and the decrease of white pulp; the green arrow shows the clear boundary of red and white pulp, and the size has no obvious change. (D) Black arrow shows a large range of necrosis of intestinal tissue, no cell structure in the necrotic area, only tissue outline and increased eosinophilic in the crypt; green arrow shows blue nuclear fragments in the necrotic tissue; blue arrow shows a small amount of inflammatory cell infiltration, such as lymphocytes; yellow arrow shows the submucosa of intestinal tissue, slightly loose and edematous. (E): Black arrow shows the structure of intestinal villi is extensively damaged, and the intestinal epithelial cells are widely exfoliated, and the lamina propria is bare; yellow arrow shows the intestinal lumen shown has many exfoliated intestinal epithelial cells; green arrow shows the some of the crypt epithelial cells are exfoliated; the exfoliated epithelial cells shown by the red arrow.
